# Comprehensive Transcriptome Analyses Reveal Candidate Genes for Variation in Seed Size/Weight During Peanut (*Arachis hypogaea* L.) Domestication

**DOI:** 10.3389/fpls.2021.666483

**Published:** 2021-05-19

**Authors:** Zhongfeng Li, Xingguo Zhang, Kunkun Zhao, Kai Zhao, Chengxin Qu, Guqiang Gao, Fangping Gong, Xingli Ma, Dongmei Yin

**Affiliations:** College of Agronomy, Henan Agricultural University, Zhengzhou, China

**Keywords:** peanut (*Arachis hypogaea* L.), transcriptome analysis, seed size/weight, pentatricopeptide repeat protein (PPR), SNP marker

## Abstract

Seed size/weight, a key domestication trait, is also an important selection target during peanut breeding. However, the mechanisms that regulate peanut seed development are unknown. We re-sequenced 12 RNA samples from developing seeds of two cultivated peanut accessions (Lines 8106 and 8107) and wild *Arachis monticola* at 15, 30, 45, and 60 days past flowering (DPF). Transcriptome analyses showed that ∼36,000 gene loci were expressed in each of the 12 RNA samples, with nearly half exhibiting moderate (2 ≤ FPKM < 10) expression levels. Of these genes, 12.2% (4,523) were specifically expressed during seed development, mainly at 15 DPF. Also, ∼12,000 genes showed significant differential expression at 30, 45, and/or 60 DPF within each of the three peanut accessions, accounting for 31.8–34.1% of the total expressed genes. Using a method that combined comprehensive transcriptome analysis and previously mapped QTLs, we identified several candidate genes that encode transcription factor TGA7, topless-related protein 2, IAA-amino acid hydrolase ILR1-like 5, and putative pentatricopeptide repeat-containing (PPR) protein. Based on sequence variations identified in these genes, SNP markers were developed and used to genotype both 30 peanut landraces and a genetic segregated population, implying that EVM0025654 encoding a PPR protein may be associated with the increased seed size/weight of the cultivated accessions in comparison with the allotetraploid wild peanut. Our results provide additional knowledge for the identification and functional research into candidate genes responsible for the seed size/weight phenotype in peanut.

## Introduction

Cultivated peanut (*Arachis hypogaea* L.), also known as groundnut, is a major cash crop with a high market value that provides both edible oil and food protein for people all over the world. At present, peanut is widely cultivated in ∼120 countries/regions with global annual production of 45.95 million tons (groundnuts, with shell) and total area harvested of 28.51 million hectares (FAOSTAT 2018^1^).

Breeding high-yielding peanut cultivars is one of the most important objectives in peanut breeding programs. A number of agronomic traits contribute to peanut yield, including height of the main stem, total branch number, and pod and seed/kernel traits (Shirasawa et al., 2012), of which the pod- and seed-related traits, such as pod size/weight and seed size/weight, directly influence final peanut production. Yield-related agronomic characters are usually complex quantitative traits that are controlled by multiple genes in crops such as rice (Song et al., 2007; Wang et al., 2015), soybean (Sun et al., 2012; Lu et al., 2017), and maize (Liu R. et al., 2012, Liu et al., 2015; Bommert et al., 2013) as well as in peanut (Huang et al., 2015; Luo et al., 2018; Zhang et al., 2019; Gangurde et al., 2020). QTL analysis has been used to identify the genomic regions that control yield-related agronomic characters in peanut. In the past decade, ≥300 QTLs for yield-related traits have been detected on all 20 peanut chromosomes (Lu et al., 2018; Zhang et al., 2019; Gangurde et al., 2020), of which 250 QTLs located on 11 chromosomes are associated with the seed and/or pod size/weight phenotype. In these linked regions, some QTLs for seed size/weight located on chromosomes 5 have been repeatedly reported across various environments in independent studies (Huang et al., 2015; Chen W. et al., 2016; Luo et al., 2017a, 2018; Lu et al., 2018; Wang Z. et al., 2018; Zhang et al., 2019; Gangurde et al., 2020). However, due to the complexity of the peanut genome structure (Bertioli et al., 2016; Yin et al., 2018; Zhuang et al., 2019) and low levels of polymorphism for molecular markers among tetraploid accessions (*A. hypogaea*) (Luo et al., 2017b, 2018), gene discovery of yield-related QTLs for seed size/weight variations has been very challenging.

Due to decreased cost and high efficiency, RNA-seq based on next-generation sequencing (NGS) technology has revolutionized genomic investigations in many plant species, and is essential for molecular marker development (Trick et al., 2012; Wang et al., 2017), mapping and identification of candidate genes for traits of interest (Liu S. et al., 2012; Islam et al., 2016; Li et al., 2019), characterization of non-coding small RNA molecules (Lu et al., 2015; Xia et al., 2019; Han et al., 2020), and detecting variations in gene structure. More importantly, in the past decade, RNA-seq has become an essential tool for the analysis of DEGs at the global transcriptome level and for the development of special gene expression atlases in various crop species (Dukowic-Schulze et al., 2014; Garg et al., 2017; Gao et al., 2018) including peanut (Chen X. et al., 2016; Clevenger et al., 2016; Sinha et al., 2020). For example, transcriptome analyses were conducted at the early and middle stages of seed maturation between wild and cultivated soybean varieties, and 2,680 DEGs were identified at these maturation stages (Lu et al., 2016). In another important legume crop, chickpea (*Cicer arientinum* L.), Garg et al. (2017) performed a comprehensive analysis of transcriptome dynamics from seven stages of seed development in two cultivars with contrasting seed size/weight, and identified key regulators that determine seed size/weight. Also, similar results have been reported by peanut researchers. For example, Chen X. et al. (2016) explored the dynamics of the peanut pod transcriptome at 11 successive developmental stages, and identified 110,217 transcripts in the pod wall. Recently, by using RNA-seq, Clevenger et al. (2016) and Sinha et al. (2020) developed a global gene expression atlas for the peanut cultivar ‘Tifrunner’ (subsp. *hypogaea* L.) and the Indian variety ICGV91114 (subsp. *fastigiata* L.), representing two subspecies of cultivated peanut (*A. hypogaea* L.), providing valuable information for functional and comparative genomics studies in groundnut.

*A. monticola*, the only allotetraploid (2*n* = 4*x* = 40) wild species in the *Arachis* genus, is considered to be either the direct progenitor of cultivated peanuts or an introgressive derivative during the evolution from diploid wild species to the allotetraploid cultivated peanut. *A. monticola* has small pods and seeds, and displays a special fruit structure different from that in *A*. *hypogaea* in that it has an isthmus separating each seed, resembling the diploid wild species (Yin et al., 2018). There are large phenotypic differences in seed traits, which are targets of interest during artificial selection, between *A. hypogaea* and *A. monticola*, but the underlying genomic changes are not well characterized.

The objective of our study was to explore the molecular mechanisms underlying seed size/weight variation between the cultivated *A. hypogaea* and its wild relative *A. monticola*. In the present work, we sequenced total extracted RNA from seeds at four different stages of development (15, 30, 45, and 60 DPF), performed comparative transcriptome analysis between two cultivated peanut accessions (Lines 8106 and 8107) and wild *A. monticola*, and also attempted to identify candidate genes located in several stable QTLs on linkage group A05 that were reported to control seed size/weight in some previous reports. The results of our study will provide useful information for further gene function research and genetic improvement of seed size/weight in peanut.

## Materials and Methods

### Plant Materials

*Arachis monticola* (PI 263393) is the only allotetraploid wild peanut among ∼80 described species in the genus *Arachis*. Line 8106 and Line 8107 (*A*. *hypogaea* L.) are groundnut lines derived from an eighth-generation recombinant inbred population, which was generated by the cross between two peanut cultivars, Huayou 7 and Huayou 4, and both have large- or medium-sized pods/seeds, with two seeds per pod. Mature seeds of *A. monticola* and Lines 8106/8107, together with twenty-seven other cultivated peanut accessions (Zp06, Yuanza9102, D7500, and so on) (Supplementary Table 1), were provided by the peanut genetics and breeding lab of Henan Agricultural University, and were planted at the experimental station of Zhengzhou, Henan, China from May to September in 2017–2019. Meanwhile, a recombinant inbred line (RIL) population (F_6_) derived from a cross of Zp06 and *A. monticola* was also planted. Row length was 2 m for all accessions; the inter-row spacing was 40 cm for Lines 8106/8107 and 90 cm for *A. monticola* because of the especially long lateral branches. Field management was the same for all groundnut accessions, and followed standard agricultural practices used in the region.

### RNA Sample Preparation

Fresh subterranean pods were harvested from 10 to 15 individual plants at 15, 30, 45, and 60 DPF and washed thoroughly with sterilized deionized water. To avoid environmental effects as much as possible, in addition to the same flowering day, the pods with roughly similar sized single-seed pods or the base pod in two-seed pods, which is adjacent to the gynophore, were considered to be at the same seed developmental stages in Line 8106 and Line 8107. Also, 15, 30, 45, and 60 DPF correspond roughly to infantile seed stage, seed expansion stage, seed-filling stage, and maturation stage, respectively. It is noteworthy that in *A. monticola*, two single-seed pods on a long gynophore are usually separated by a short isthmus, which is apparently different from cultivated peanuts (Yin et al., 2018), and the pod farthest from the peg tip was selected for RNA extraction since it resembles the base pod in Line 8106/8107 from the perspective of seed development. For each stage, enough seeds were quickly collected, immediately frozen in liquid nitrogen, and stored at −80°C for use in the subsequent research.

For each of the four seed developmental stages in Line 8106, Line 8107, and *A*. *monticola*, 10–20 seeds were pooled and ground to a powder and mixed well for RNA extraction. Total RNA was extracted using an RNA Prep Pure Plant kit (Tiangen Co., Beijing, China), and treated with DNaseI (Thermo Fisher Scientific Inc., Grand Island, NY, United States) to remove contaminating genomic DNA. A total of 12 RNA samples were initially checked by agarose gel electrophoresis (1%, w/v), and the RNA integrity and purity were then analyzed using the RNA Nano 6000 Assay Kit on the Agilent Bioanalyzer 2100 system (Agilent Technologies, CA, United States), and concentrations were estimated using the NanoPhotometer^®^ spectrophotometer (IMPLEN, CA, United States). In addition, the Qubit^®^ RNA Assay Kit on the Qubit2.0^®^ Flurometer (Life Technologies, CA, United States) was also used to further measure RNA concentration of all samples, and those with RIN number ≥ 7 were prepared for RNA-seq library construction.

### RNA-Seq Library Construction and Illumina Sequencing

According to the manufacturer’s instructions (Illumina Inc.), ∼3-μg RNA of each of the 12 RNA samples was used to generate the sequencing libraries using NEBNext^®^ Ultra^TM^ RNA Library Prep Kit for Illumina (New England Biolabs, United States), and index codes were added to attribute sequences of the individual samples. Briefly, mRNA isolation, fragmentation, synthesis of double strand cDNA, end modifications (i.e., adenylation, adaptor ligation), fragment selection, PCR reactions, and amplification product purification (AMPure XP system) were performed in succession. The quality of each library (insert size length and effective concentration ≥ 2nM) was strictly assessed on the Agilent Bioanalyzer 2100 system, and the paired-end sequencing libraries with an insert size of approximately 270 bp were sequenced on an Illumina HiSeq X ten instrument, and paired-end reads with the length of 150 nucleotides were generated.

### RNA-Seq Data Processing, Gene Annotation, and SNP Calling

Huge numbers of raw reads were generated from the 12 RNA samples derived from the three peanut accessions. High-quality clean reads were obtained by removing low-quality reads with ≥10% ambiguous nucleotides (Ns), or ≥50% bases in which the quality scores (*Q*-score) were ≤10, as well as excluding reads that contained adapter sequences. In addition, the percentages of reads with *Q*-scores of Q20 and Q30, and the GC contents and sequence duplication level of the clean reads were also estimated. The clean reads were then aligned to a *de novo* genome assembly of the allotetraploid wild peanut *A. monticola* (Yin et al., 2018) using HISAT2 (Kim et al., 2015), which is highly efficient at aligning reads from RNA-seq experiments. Transcripts were assembled using StringTie (Pertea et al., 2015) while gene expression levels were quantified by applying the fragments per kilobase per million reads (FPKM) method of RSEM (Li and Dewey, 2011). The annotations of gene function for the assembled transcripts were then performed based on searches of multiple databases, including Nr (NCBI non-redundant protein sequences), Nt (NCBI non-redundant nucleotide sequences), Pfam (Protein family), KOG/COG (Clusters of Orthologous Groups of proteins), Swiss-Prot (a manually annotated and reviewed protein sequence database), KO (KEGG Ortholog database), and GO. Variant calling and annotation was conducted following the protocol of Zhou et al. (2015) with slight modification. GATK2 (McKenna et al., 2010) or Samtools (Li et al., 2009) software was used to perform SNP calling. Raw VCF files were filtered with GATK standard filter method and other parameters (cluster Window Size: 10; MQ0 ≥ 4 and (MQ0/(1.0^∗^DP)) > 0.1; QUAL < 10; QUAL < 30.0 or QD < 5.0 or HRun > 5), and only SNPs with distance >5 were retained.

### Differential Gene Expression, GO and KEGG Pathway Enrichment Analyses

Because each of the 12 samples from Lines 8106, 8107, and *A*. *monticola* were composed of 10–20 pooled individual seeds at the same seed development stage, identification of DEGs between pairs of the 12 samples was performed using the EBSeq R package, and only those genes with both fold-change ≥ 2 and false discovery rate (FDR) < 0.01 were considered to be differentially expressed (Leng et al., 2013). Subsequent GO enrichment tests of the DEGs were performed by using the GOseq R package based on the Wallenius non-central hyper-geometric distribution (Young et al., 2010). We used KOBAS (Mao et al., 2005) to perform KEGG pathway enrichment for the resulting DEGs between the two cultivated peanut accessions and the tetraploid wild relative.

### Stage-Specific Gene Expression and GO Enrichment Analyses

A gene was defined as being specifically expressed when expression was only detected in one of the four seed developmental stages. Genes with stage-specific expression were identified by creating a Venn diagram of all expressed genes at the four stages for each of the three peanut accessions (Line 8106, Line 8107, and *A*. *monticola*) (Oliveros, 2007–2015), and the corresponding GO annotations were conducted as mentioned above.

### Identification of Candidate Genes Responsible for Variation in Seed Size/Weight

Candidate physical intervals containing the seed size/weight QTLs were determined when the corresponding flanking markers were mapped on the chromosomal pseudomolecules of *A. monticola* using BLAST. Combined with the identification of DEGs and sequence variation detection, the sequences within these candidate physical chromosomal regions were further used to screen for the potential candidate genes underlying the targeted QTLs for seed size/weight variations between cultivated peanut and the tetraploid wild relative.

### DNA Extraction, SNP Marker Development, and Genotyping of Peanut Accessions

A young leaf was collected from each of peanut accessions at the seedling stage. Genomic DNA was extracted using a modified CTAB method (Saghai-Maroof et al., 1984), and diluted to a concentration of 50 ng/μL in ddH_2_O. The markers were developed as follows. The clean reads for transcriptome from *A. monticola*, Line 8107, and Line 8106 were aligned to the *A. monticola* (PI 263393) genome assembly (Yin et al., 2018), respectively, and SNP calling was performed as above. Furthermore, by comparing the two cultivated lines to *A. monticola*, the overlapped SNP variations were identified in both Line 8106 and Line 8107. Based on the previously mapped QTLs and GO/KEGG enrichment, the genes with SNP variations were considered as potential candidates for further analysis. After downloading the corresponding genomic sequence from the *A. monticola* genome assembly (Yin et al., 2018), special primers were designed online using Primer3^2^, and the amplified PCR fragments were sequenced using the Sanger method (Sanger et al., 1977) for SNP identification. The developed SNP markers for each of candidates were then used to genotype thirty peanut accessions including *A. monticola*, Line 8016 and Line 8107, as well as 62 RILs from a cross of Zp06 × *A. monticola* (Supplementary Table 1). PCR reactions (30 μL) consisted of 2 μL genomic DNA (100 ng), 15 μL Phanta max buffer (2×), 0.5 μL dNTP mix (10 mM each), 1 μL forward and reverse primers (10 pmol), 0.5 μL Phanta max super-fidelity DNA polymerase (P505-d1, Vazyme, China) and sterile water. PCR amplification started with a denaturing step at 94°C for 3 min, followed by 36 cycles of denaturation at 95°C for 30 s, annealing at 57°C for 40 s, and extension at 72°C for 40 s, with a final extension step at 72°C for 10 min. PCR products were separated on 1.5% agarose gels, stained with ethidium bromide, visualized on a UV transilluminator, and then purified and sequenced on the Sanger sequencing platform (Sanger et al., 1977).

### Data Availability

All raw sequences for transcriptome are available in the NCBI Sequence Read Archive under accession numbers: SRR8334380, SRR8334357, SRR8334346, SRR8334385 for *A. monticola* 15–60 DPF; SRR8334375–SRR8334377, SRR8334345 for Line 8107 15–60 DPF; SRR8334371–SRR8334374 for Line 8106 15–60 DPF, respectively.

## Results

### Phenotyping Seed Size/Weight in Line 8106, Line 8107, and *A. monticola*

**FIGURE 1:**
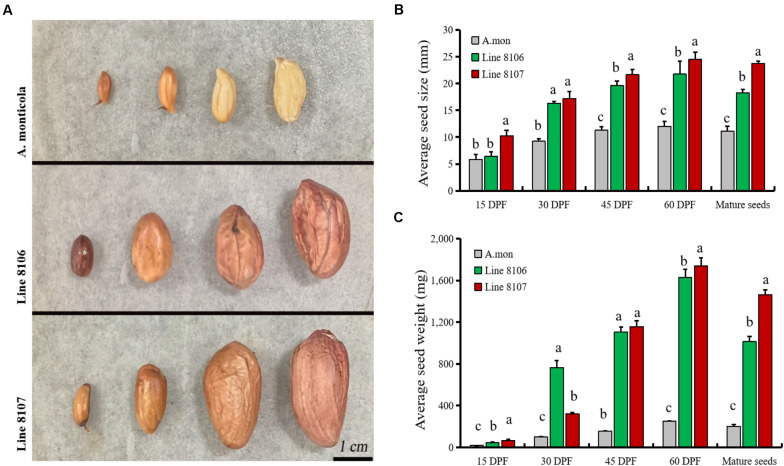
Seed phenotypes of three peanut accessions at four seed developmental stages. **(A)** Seed samples at 15, 30, 45, and 60 DPF from *A. monticola* (*A. mon*), Line 8106, and Line 8107, scale bar = 1 cm. Panels **(B,C)** show the changes in average size and weight for seeds from *A. mon*, Line 8106, and Line 8107 from 15 DPF to maturity. One-way analysis of variance was performed. Significant level, α = 0.05.

Large variations in agronomic traits are observed in plant type as well as pod/seed traits between the two cultivated peanut accessions and the tetraploid wild *A. monticola*. It is well known that seed size/weight directly determines the final yield in peanut. In this study, fresh representative pods from Line 8106, Line 8107, and *A. monticola* at 15, 30, 45, and 60 DPF (near the end of seed development) were collected and measured (Figure 1A). The results showed that seed size/weight in Lines 8106 and 8107 was significantly higher than that in the tetraploid wild relative from 30 to 60 DPF, while a similar seed size was observed among the three peanut accessions at 15 DPF (Figures 1B,C), suggesting that genetic factors contribute to seed size/weight variations between cultivated and wild tetraploid peanut accessions.

### Sequencing Data for the Two Cultivated Accessions, Line 8106 and Line 8107, and the Wild Peanut *A. monticola*

**FIGURE 2:**
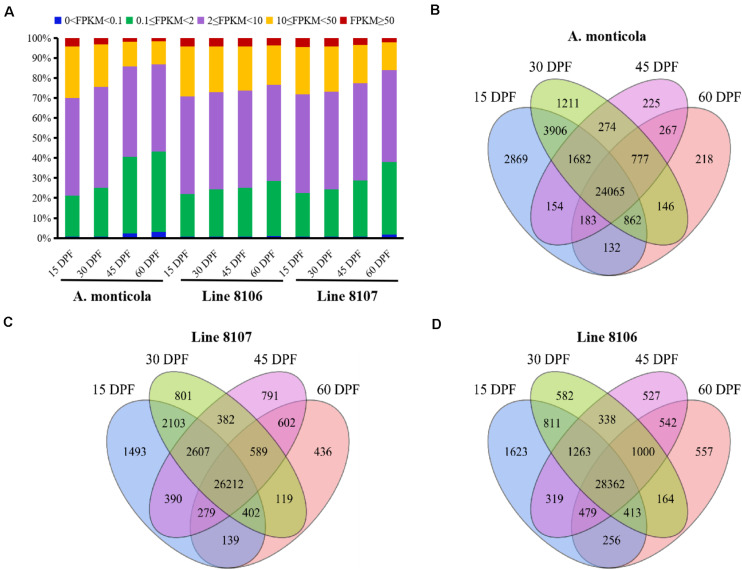
Expressed genes in the three peanut accessions at four seed developmental stages. **(A)** Proportion of genes expressed at five different levels (based on FPKM) during the four seed developmental stages (15, 30, 45, and 60 DPF) for *A. monticola*, Line 8106, and Line 8107. Panels **(B–D)** are Venn diagrams showing the number of genes with shared or stage-specific expression at the four seed developmental stages (15, 30, 45, and 60 DPF) in *A. monticola*, Line 8107, and Line 8106, respectively.

To investigate the transcriptome profile during peanut seed development, we sequenced 12 RNA samples extracted from developing seeds at 15, 30, 45, and 60 DPF of two cultivated peanut accessions, Line 8106 and Line 8107, and the tetraploid wild species *A. monticola*. A total of 97.8, 93.6, and 96.3 million high-quality clean paired-end reads with Q30 ≥ 88.9% were generated for Line 8106, Line 8107, and *A. monticola* for the four developmental stages (∼24 million clean reads for each sample or stage per accession on average), respectively (Table 1). We found that 83.76%–92.09% of these clean reads were mapped to unique or multiple locations in the *A. monticola* reference genome. Approximately 36,000 gene loci were expressed in each of the 12 developmental stages or samples consisting of 30,105–31,076 known and 5,310–5,724 novel gene loci accounting for 84.2–85.1% and 14.9–15.6% of the genes, respectively, of which ∼95% were functionally annotated by searches of several databases including COG (Clusters of Orthologous Groups), GO, KEGG, Swiss-Prot, eggNOG (evolutionary genealogy of genes: Non-supervised Orthologous Groups), and Nr (Figure 2 and Supplementary Tables 2, 3).

**TABLE 1:** Summary of RNA-seq data for 12 seed samples from Line 8106, Line 8107 and *A. monticola* at 15, 30, 45, and 60 days past flowering.

Materials	Samples	Number of total clean reads^a^	Number of mapped reads^b^ and percentage^c^ (%)	GC content (%)	≥Q30%
*A. monticola*	T01 (15 DPF)	23,032,880	41,101,117 (89.22)	44.46	92.03
	T02 (30 DPF)	26,328,402	44,119,348 (83.79)	45.64	91.83
	T03 (45 DPF)	22,558,229	39,038,896 (86.53)	47.81	91.55
	T04 (60 DPF)	24,553,778	45,222,243 (92.09)	47.42	91.67
Line 8106	T19 (15 DPF)	20,484,678	35,316,212 (86.20)	45.39	88.92
	T20 (30 DPF)	23,833,288	41,594,709 (87.26)	45.09	89.87
	T21 (45 DPF)	25,071,427	44,010,908 (87.77)	45.31	89.39
	T22 (60 DPF)	28,697,647	50,498,677 (87.98)	45.74	89.82
Line 8107	T23 (15 DPF)	22,591,178	37,843,394 (83.76)	44.53	91.99
	T24 (30 DPF)	25,811,138	45,169,166 (87.50)	45.14	89.91
	T25 (45 DPF)	22,632,401	39,734,238 (87.78)	46.02	89.62
	T26 (60 DPF)	22,771,158	40,371,333 (88.65)	47.11	89.35

*^*a*^The number of paired-end reads generated for each sample.^*b*^The number of those single-end reads mapped on the reference genome of *A. monticola*.^*c*^The total number of single-end reads generated divided by the number of those single-end reads mapped on the reference genome for each sample × 100.*

To further examine genome-wide gene expression patterns, the expressed genes were divided into five classes (very high, FPKM ≥ 50; high, 10 ≤ FPKM < 50; moderate, 2 ≤ FPKM < 10; low, 0.1 ≤ FPKM < 2; and very low, 0 < FPKM < 0.1) based on the number of FPKM for each transcript. Overall, nearly half of the detected genes exhibited moderate (2 ≤ FPKM < 10) expression levels in all of the 12 samples analyzed (Figure 2A). With seed development, the number of genes showing high (FPKM ≥ 10) expression levels dropped dramatically (e.g., from 29.8% at 15 DPF to 13.3 at 60 DPF in *A. monticola*), while the number of low-expression genes (0 < FPKM < 2) substantially increased (e.g., from 21.2% at 15 DPF to 43.3% at 60 DPF in *A. monticola*). Also, we found that comparing 45 to 30 DPF, the ratio of high-expression genes was reduced dramatically (from 24.4% to 14.4%) and the ratio of low-expressed genes showed a sharp increase (from 25.1% to 40.6%) in *A. monticola*. However, the similar situation probably occurred at a later seed developmental stage, namely between 45 DPF and 60 DPF, in both Lines 8106 and 8107. These results suggest that substantial genome-wide changes in gene expression occur at different seed development stages, and this could be associated with the observed seed size variation between tetraploid wild *A. monticola* and the cultivated peanut accessions.

### Stage-Specific Genes Expressed During Seed Development in Line 8106, Line 8107, and *A. monticola*

A total of ∼36,000 genes were found to be expressed in the developing peanut seeds at four stages of development (15, 30, 45, and 60 DPF) in Line 8106, Line 8107, and *A. monticola*. Of these, 65.1% (24,065) were expressed simultaneously across the four stages, and 12.2% (4,523) were specifically expressed at one of the four seed developmental stages in *A. monticola* (Figure 2B). While there was a minor increase in the number of concurrently expressed genes among the four stages, a slight reduction in the number of specifically expressed genes was observed in both cultivated peanut accessions (Figures 2C,D). Moreover, stage-specific genes were expressed mainly at 15 DPF, and the number of these genes then decreased gradually with advancing seed development in all three peanut accessions.

To preliminarily explore what kinds of genes are expressed in a stage-specific manner, GO enrichment analyses were performed at the four stages of seed development for Line 8106, Line 8107, and *A. monticola*. The results revealed that various biological process terms such as ‘cell cycle phase,’ ‘DNA replication,’ ‘cell stress responses,’ ‘protein folding and modification,’ ‘pigment biosynthetic/metabolic process,’ ‘transmembrane transport,’ and ‘signaling pathway’ were enriched. In particular, the genes associated with the ‘cell cycle phase’ and ‘DNA replication’ were mainly expressed at 15 DPF in *A. monticola*, while their counterparts were preferentially enriched at 30 and 45 DPF in both Line 8106 and Line 8107. In addition, enrichment of the GO term ‘carbohydrate metabolic processes’ was quite different between *A. monticola*, Line 8106, and Line 8107 because the related genes were only enriched at 15 DPF in wild *A. monticola*, whereas their counterparts were consecutively expressed at three seed developmental stages except for 60 DPF in the two cultivated peanut accessions (Supplementary Tables 9–11).

### Differential Gene Expression Between the Four Seed Developmental Stages in the Individual Peanut Accessions

**FIGURE 3:**
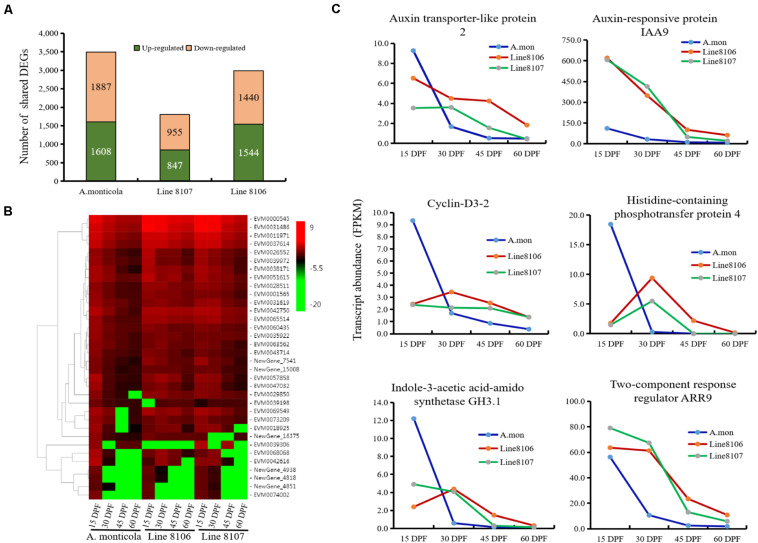
Differential gene expression at the four seed developmental stages in each of the three peanut accessions. **(A)** The number of up- and down-regulated genes shared among three seed developmental stages (30, 45, and 60 DPF) for *A. monticola*, Line 8106, and Line 8107. **(B)** Heatmap showing the relative expression of plant hormone signaling pathway-related DEGs. **(C)** Expression levels of six representative genes from 15 to 60 DPF that are enriched in the plant hormone signaling pathway.

To study the factors that contribute to increased seed size during seed development, differential gene expression analysis between the different stages (15 DPF vs. 30 DPF, 15 DPF vs. 45 DPF, and 15 DPF vs. 60 DPF) was performed in Line 8106, Line 8107, and *A. monticola*, respectively. When compared to 15 DPF, a total of ∼12,000 genes were differentially expressed at 30, 45, and/or 60 DPF for each of the three peanut accessions, accounting for 31.8–34.1% of the total number of expressed genes (Supplementary Table 5). We also found that DEGs predominated at 60 DPF for all of three peanut accessions. Additionally, some genes were shared by the three DEG sets, e.g., 3,495 genes exhibited differential expression at 30, 45, and 60 DPF in *A. monticola*, while fewer DEGs were identified concurrently in both cultivated peanut accessions, with 1,802 and 2,984 down-/up-regulated genes for Line 8107 and Line 8106, respectively (Figure 3A).

The up-/down-regulated genes shared by the three DEG sets may contribute to increased seed size during seed development, so we performed GO enrichment analysis. The results showed that some GO terms such as ‘carboxylic/organic acid biosynthetic process,’ ‘thiazole biosynthetic process,’ ‘protein synthesis and modification,’ and ‘small molecules metabolism process (thiamine biosynthetic process and flavonoid metabolic process)’ were significantly represented in the up-regulated genes shared by *A. monticola*, Line 8106, and 8107, and the term ‘pigment metabolic process’ was concurrently enriched in the shared down-regulated genes (Supplementary Table 4). Notably, the ‘cell division’- or ‘regulation of cell morphogenesis’-related GO term was represented in the down-regulated genes common to the three DEG sets for *A. monticola* and the cultivated peanut Line 8106, respectively (Figure 3C, Supplementary Table 4), while the ‘mitotic G2 phase’ GO term was significantly enriched in the up-regulated genes shared by the three DEG sets in Line 8107. We also found that the ‘stress response’ GO term was represented in the two cultivated peanut accessions, while the term ‘seed dormancy’ was only significantly enriched in *A. monticola*. In addition, another GO term, ‘gibberellin metabolic process or GA-mediated signaling pathway’ was only enriched in the DEGs shared among the three DEG sets from the cultivated accession Line 8106 and the wild peanut (Supplementary Table 4).

### Differential Expression of Genes During Seed Development Between the Tetraploid Cultivars and the Wild Peanut

**FIGURE 4:**
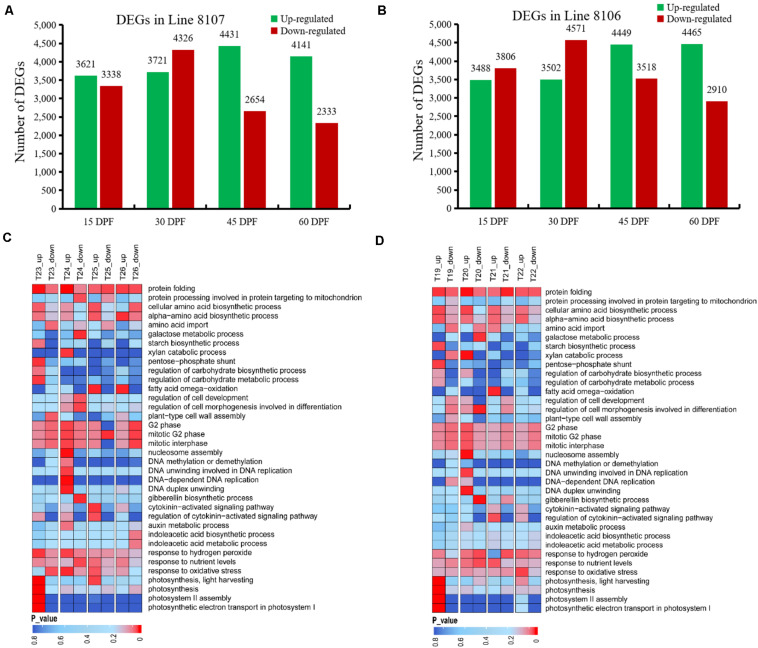
DEGs between *A. monticola* and two cultivated peanut accessions at four seed developmental stages. Up-/down-regulated genes were detected in Line 8107 **(A)** and Line 8106 **(B)** at 15, 30, 45, and 60 DPF in pairwise comparisons with the wild peanut species *A. monticola*. Some GO terms were significantly enriched in the DEGs from Lines 8107 and 8106, and are shown in panels **(C,D)**, respectively. T19–T22, and T23–T26 indicate the seed samples at 15, 30, 45, and 60 DPF from Line 8106 and Line 8107, respectively.

To investigate candidate genes involved in seed size variation, we next performed differential gene expression analysis between *A. monticola* and Lines 8106/8107 for each of the four stages of seed development. When compared to the wild peanut *A. monticola*, we identified 16,118 genes that exhibited differential expression across the four seed developmental stages (15, 30, 45, and 60 DPF) in Line 8107, of which there were 8,047 DEGs at 30 DPF followed by 7,085, 6,959, and 6,474 DEGs at 45, 15, and 60 DPF, respectively (Figure 4A). Of these DEGs, 4,431 genes showed higher expression levels at 45 DPF with more up-regulated genes than in the other stages in the peanut accession Line 8107. Interestingly, a similar trend was also observed in Line 8106, in which a total of 16,802 DEGs were detected across all four stages (Figure 4B), whereas 4,449 and 4,465 genes, an almost equal number, were up-regulated at 45 and 60 DPF, respectively.

The GO term analyses were further conducted on four DEG sets (15DPF-*A.mon*_vs_8107, 30DPF-*A.mon*_vs_8107, 45DPF-*A.mon_*vs_8107 and 60DPF-*A.mon_*vs_8107) between *A. monticola* and Line 8107. Various GO terms were enriched including ‘cell cycle phase and cell wall assembly,’ ‘carbohydrate biosynthetic/metabolic process,’ ‘photosynthesis,’ ‘DNA replication and modification,’ ‘protein biosynthesis,’ ‘maintenance of seed dormancy,’ ‘auxin metabolic process,’ and ‘gibberellin biosynthetic process,’ and others (Figure 4C). The G2 phase of mitotic cell cycle-related genes had lower expression levels at both 15 and 45 DPF; however, they showed higher expression levels at 30 DPF in Line 8107 (Figure 4C and Supplementary Figure 3). Similarly, the GO terms ‘nucleosome assembly’ and ‘DNA unwinding involved in DNA replication and DNA replication initiation’ were also highly enriched in the genes showing up-regulated expression at 30 DPF (Figure 4C and Supplementary Figure 3). Additionally, several biological processes associated with phytohormone metabolism, such as ‘auxin metabolic process,’ ‘regulation of hormone levels,’ and ‘cytokinin-activated signaling pathway,’ as well as ‘tryptophan biosynthetic process,’ were also identified and found to be enriched mainly in the up-regulated genes at 30 and/or 45 DPF in Line 8107, while some genes related to ‘gibberellin biosynthetic process’ showed lower expression levels at 30 DPF (Figure 4C and Supplementary Figure 3). In addition, some other GO terms, like ‘photosynthesis,’ ‘regulation of carbohydrate metabolic process,’ and ‘protein folding,’ were uniquely/commonly over-represented at different stages (15–60 DPF) of seed development, and the related genes mainly showed significantly higher expression levels in the cultivated peanut Line 8107. Maintenance of seed dormancy-related genes seemed to be less active at 15 DPF, while they were up-regulated at 30 DPF (Figure 4C and Supplementary Figure 3).

A similar situation was also observed in Line 8106 (Figure 4D and Supplementary Figures 1–3). For example, the ‘G2 phase of mitotic interphase’ was also over-represented in some down-regulated genes at 15 DPF, encoding mitochondrial import inner membrane translocase subunit and 26S proteasome regulatory subunit. At 30 DPF, 19 genes that exhibited higher expression levels were concurrently enriched in the ‘nucleosome assembly’ term in both Line 8107 and Line 8106; these genes encode histones H2A, H2B, H3, and H4 (Supplementary Figure 2). Moreover, genes associated with the terms ‘DNA-dependent DNA replication’ and ‘DNA modification’ also showed higher expression levels in the two cultivated peanut accessions. Of these, the former include DNA replication licensing factor, 125 kDa kinesin-related protein, and cell division control protein 45, and genes for DNA (cytosine-5)-methyltransferase 1, ATP-dependent DNA helicase DDM1-like, and protein argonaute 4A-like were included in the latter term (Supplementary Figure 3). At 45 DPF, we found that some up-regulated genes were simultaneously engaged in the ‘cytokinin-activated signaling pathway’ in both of the cultivated lines and they encode alpha-methyl-mannoside-specific lectin and histidine-containing phosphotransfer protein 1, and other proteins (Supplementary Figure 3). These results show that the shared GO terms and related genes may play an important role in regulating seed development in peanut.

### Screening for Candidate Genes and SNPs Associated With Seed Size/Weight Variations Differing Between the Tetraploid Wild Peanut and Its Cultivated Relatives

Some GO terms, such as ‘cell division’ and ‘pigment biosynthesis,’ were enriched in the genes that showed differential expression between the different seed development stages (15 DPF vs. 30 DPF, 15 DPF vs. 45 DPF and 15 DPF vs. 60 DPF) in Line 8106, Line 8107, and *A. monticola*. Coincidentally, these terms were also identified in some DEGs between *A. monticola* and Lines 8106/8107 at four stages of seed development. Therefore, we hypothesize that these GO terms, especially for the cell division-related genes, may be closely related to seed size variation during seed development within and between peanut accessions.

In *A. monticola*, a total of 16 cell division-related GO terms including ‘nucleosome assembly,’ ‘microtubule-based process,’ ‘cytokinesis,’ ‘cytoskeleton-dependent cytokinesis,’ ‘cellular component movement,’ ‘microtubule-based movement,’ ‘microtubule cytoskeleton organization,’ ‘mitotic chromosome condensation,’ and ‘mitotic G2 phase,’ were over-represented in numbers of down-regulated genes shared at 30, 45, and/or 60 DPF (Supplementary Table 4). Of these genes, 21 were concurrently shared by several terms such as ‘microtubule cytoskeleton organization,’ ‘cytokinesis’ and ‘mitotic G2 phase,’ encoding kinesin-like protein, serine/threonine-protein kinase, laccase/diphenol oxidase family protein, protein COBRA, and glucuronoxylan glucuronosyltransferase based on the Nr_annotation (Supplementary Figure 1). Thirty-one other genes that encode histones H1, H2A, H2B, H3, and H4 were only involved in nucleosome assembly. Both types of genes exhibited gradually decreased expression levels with advancing seed development; however, a sharp decline in FPKM values was observed at 45 or 60 DPF in the cultivated peanut accession Line 8106 or Line 8107 rather than at 30 DPF as in *A. monticola*, suggesting that these genes may be related to peanut seed development (Supplementary Figure 2).

**FIGURE 5:**
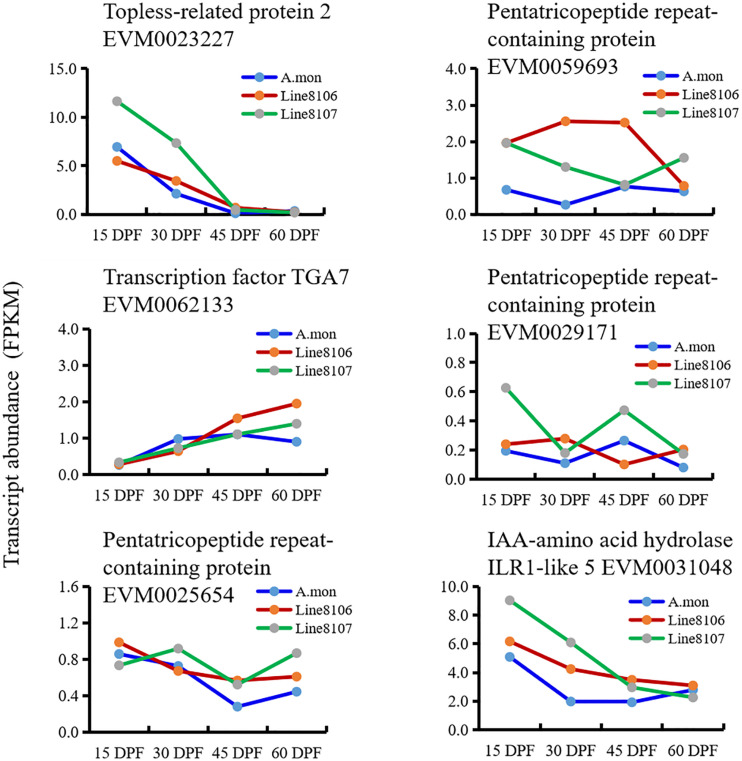
Expression levels of identified candidate genes associated with peanut seed size/weight in *A. monticola* (*A. mon*), Line 8106, and Line 8107. The genes EVM0023227, EVM0062133, and EVM0031048 encode topless-related protein 2, transcription factor TGA7, and IAA-amino acid hydrolase ILR1-like 5, respectively. The other genes encode pentatricopeptide repeat-containing proteins.

To explore the processes that contribute to seed size variation during seed development, all genes enriched in 16 cell division-related GO terms were further analyzed in some detail. The results showed that three homozygous SNPs (T>A, C>T, and G>T at nucleotide positions 4381965, 4384056, and 4384999, respectively) are located in the coding region of the gene EVM0023227, resulting in amino acid variations in the sequence of topless-related protein 2. Based on the GO enrichment analyses, EVM0023227 is involved in ‘reproductive process’ (GO:0022414), ‘microtubule cytoskeleton organization’ (GO:0000226), and ‘cytokinesis by cell plate formation’ (GO:0000911) in the ‘biological process’ GO category (Table 2). Furthermore, KEGG enrichment analyses were also performed on the DEGs that were shared among the three seed developmental stages in *A. monticola* (Figure 3B). EVM0062133, which encodes transcription factor TGA7, participates in the plant hormone signal transduction pathway, and has two homozygous SNPs (A>C, T>A at nucleotides 34969001 and 34969010, respectively) in the coding sequence leading to changes in the amino acid sequence in both of the cultivated peanut accessions (Table 2). In addition, we also determined transcript abundance for the two genes EVM0023227 and EVM0062133 (Figure 5). With advancing seed development, expression levels decreased steadily for the topless-related protein 2-encoding gene EVM0023227 in all three peanut accessions, and the highest relative transcript levels were in Line 8107 at 15 and 30 DPF. mRNA levels for the EVM0062133 gene showed significant increases during seed development in all three peanut accessions, and the highest relative expression levels were detected at 45 and 60 DPF in the two cultivated peanut accessions.

**TABLE 2:** Summary of identified candidate genes related to the seed size/weight phenotype based on RNA-seq and QTL mapping.

Gene_ID	Chromosome^a^	Physical position (bp)	References^b^	Variation^c^	SNP effect	Codon change	Predicted gene function^d^	Biological process^e^
EVM0023227	A.mon-A04	4381965	T	A	Non-synonymous	gaA → gaT	Topless-related protein 2	Developmental process (GO:0032502), reproductive process (GO:0022414)
		4384056	C	T		aGa → aAa		
		4384999	G	T		Ctg → Atg		
EVM0062133	A.mon-A04	34969001	A	C	Non-synonymous	aTt → aGt	Transcription factor TGA7	Biological regulation (GO:0065007)
		34969010	T	A		aAc → aTc		
EVM0027962	A.mon-A05	109865939	T	C	Non-synonymous	gAt → gGt	Uncharacterized protein	–
EVM0025654	A.mon-A05	111201604	C	G	Non-synonymous	tGt → tCt	Pentatricopeptide repeat-containing protein	–
EVM0059693	A.mon-A05	111487529	G	C	Non-synonymous	gGg → gCg	Pentatricopeptide repeat-containing protein	–
EVM0031048	A.mon-B10	103762383	C	T	3′ UTR	–	IAA-amino acid hydrolase ILR1-like 5	Cellular process (GO:0009987), biological regulation (GO:0065007)

*^*a,b*^Indicate the assembled chromosomes and the genotypes of the SNP loci in the *A. monticola* reference genome, respectively.^*c*^Represents the genotypes of the corresponding locus present in the genomes of the cultivated peanut accessions Lines 8106 and 8107.^*d*^Gene function was predicted based on searches of the Swiss_Prot and Nr databases.^*e*^The involved biological process is based on the GO enrichment analysis.*

Similar analyses were also performed on the DEGs between *A. monticola* and Lines 8106/8107. Although some GO terms such as ‘G2 phase of mitotic interphase,’ ‘nucleosome assembly,’ ‘DNA-dependent DNA replication and DNA modification,’ and ‘cytokinin-activated signaling pathway’ were concurrently enriched in the up- and down-regulated genes in both Line 8107 and Line 8106, no SNPs resulting in non-synonymous changes in amino acid sequence were identified in the genomic sequences of the genes in the two cultivated peanut accessions. However, EVM0031048, which encodes IAA-amino acid hydrolase ILR1-like 5, is enriched in the GO term ‘regulation of hormone levels,’ and has the homozygous SNP C>T (at nucleotides 103762383 on chromosome A.mon-B10) in the 3′ untranslated regions (UTR) of the coding sequence in both of the cultivated peanut accessions (Table 2). In addition, EVM0031048 showed elevated expression levels in Lines 8106 and 8107 from 15 DPF to 45 DPF (Figure 5).

Overall, these results suggest that EVM0023227, EVM0062133, and EVM0031048 may be related to seed size/weight variations between tetraploid wild *A. monticola* and cultivated peanut accessions Line 8106 and Line 8107.

### Identification of Candidate Genes Underlying the QTLs for Seed Size/Weight Based on RNA-Seq Analyses

Some QTLs for seed size/weight on linkage group A05 have been repeatedly detected by previous studies (Supplementary Table 6). Accordingly, the underlying candidate genes for seed size/weight variation may act independently of the environment and genetic background. After basic information on the QTLs was collected, analyzed, and integrated successively, two genomic regions associated with seed size/weight, designated *Region1* and *Region2*, were found to be located adjacent to one another on the A05 pseudomolecule (A.mon_A05) of the *A. monticola* genome assembly, and correspond to physical intervals of ∼3.02 Mb (115.52–118.54 Mb) and ∼7.40 Mb (107.44–114.86 Mb), respectively.

We then analyzed the RNA-seq data from the three peanut accessions, and six homozygous SNPs were detected in *Region2*, while for *Region1* only one and two homozygous SNPs were identified in the genomes of Line 8107 and Line 8106, respectively (Supplementary Table 7). Of these SNPs, three exist simultaneously in *Region2* in the two cultivated peanut accessions, which result in amino acid sequence variations of EVM0027962 (T to C at nucleotide 109865939, uncharacterized protein), EVM0025654 (C to G at nucleotide 111201604, pentatricopeptide repeat-containing protein), and EVM0059693 (G to C at nucleotide 111487529, pentatricopeptide repeat-containing protein) (Table 2 and Supplementary Table 7). In *Region1*, the SNP A>T at nucleotide 119625225 shared by Line 8106 and Line 8107 is located in the 3′ untranslated region of EVM0074021, a gene that encodes an unknown protein. In addition, RNA-seq data from another peanut accession HuaX (medium-size seed/pod) was also analyzed (unpublished data), and both the C>G transversion at nucleotide 111201604 in EVM0025654 and the A>T transversion at nucleotide 119625225 in EVM0074021 were identified in *Region1* and *Region2*. We also further evaluated the transcript abundance of these genes with SNP variations located in the two linked mapping intervals (Figure 5). These results show that compared to *A. monticola*, none of these genes were differentially expressed except for EVM0059693 and EVM0029171 in the two cultivated peanut accessions. Interestingly, half of these 10 genes encode pentatricopeptide repeat-containing proteins including EVM0059693, EVM0029171, EVM0058772, EVM0029388, and EVM0025654 (Supplementary Table 7), suggesting that SNP variations in the genomic sequences or differential expression levels of the genes encoding pentatricopeptide repeat-containing proteins may be associated with seed size/weight variations between cultivated peanut accessions and the tetraploid wild relative *A. monticola*.

### Validation of Candidate SNPs and Their Association With Increased Seed Size/Weight in Cultivated Peanut Accessions

**FIGURE 6:**
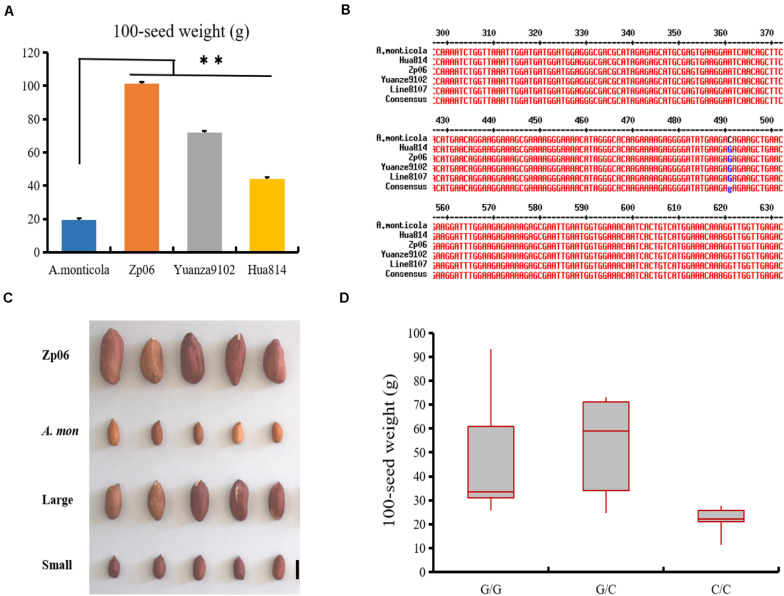
EVM0025654 is associated with increased seed size/weight in cultivated peanut. **(A)** 100-seed weights for *A. monticola* and three cultivated peanut accessions. **(B)** The marker SNP564 was used to genotype these cultivated peanut lines and the SNP locus at position 490 is shown in blue. **(C)** Seeds of recombinant inbred line (RIL) population (F_6_) derived from a cross of Zp06 (100-seed weight, 106.2 g) and *A. monticola* with a 100-seed weight of 19.4 g. Large, some RILs have large seeds; small, others lines have small seeds. **(D)** Box plot for seed weight of the RILs having G/G, G/C, and C/C genotype at the SNP locus (111201604 bp) in EVM0025654. A Student’s *t*-test indicated a significant difference (*n* = 5 plants) in panel **(A)**. ^∗∗^*P* < 0.01. All data are given as mean ± SD. Bar = 10 mm.

Several SNP markers were developed based on sequence variations identified from the analysis of RNA-seq data (Supplementary Table 8). Using PCR amplification and DNA sequencing, we found that the SNP variations in EVM0027962 and EVM0074021 were not present in the genes from Line 8107 or Line 8106, while other SNPs in EVM0023227, EVM0025654, and EVM0031048 were validated in both cultivated accessions. Furthermore, the marker SNP654 was used to genotype twenty-seven other cultivated peanut accessions with 100-seed weight of 28–118 g including Zp06, Yuanza9102, D7500, and so on (Figure 6A and Supplementary Table 1). The results showed that all of them were G/G at the SNP locus (111201604 bp) in EVM0025654 on chromosome A.mon-A05 (Figure 6B). However, the C/C genotype was only detected at the corresponding locus in *A. monticola* with a 100-seed weight of 19.4 ± 1.8 g (Figure 6B), implying that EVM0025654, a gene that encodes a pentatricopeptide repeat-containing protein, may be partly responsible for the increased seed size/weight that occurred during the domestication of the cultivated peanut from its wild ancestor. To further test this possibility, we also genotyped a recombinant inbred line (RIL) population (F_6_) derived from a cross of Zp06 (100-seed weight, 106.2 g) and *A. monticola* using the SNP654 marker. All of 20 lines with homologous G/G or heterozygous G/C genotype at the target SNP locus show bigger seed size and weight (100-seed weight of 26–93.2 g) compared to *A. monticola* (*P* < 0.01), while 18 out of 21 lines showing homologous C/C genotype at the corresponding locus have a similar seed phenotype to the wild parent (Figures 6C,D). Taken together, the results strongly indicated that allelic variation in EVM0025654 is associated with the increased seed size/weight of the cultivated peanut accessions in comparison with the allotetraploid wild peanut.

## Discussion

### Peanut Seed Size/Weight Is an Important Domestication-Related Target

Various agronomic traits have been subjected to artificial or natural selection during crop domestication, and understanding the underlying molecular mechanisms will facilitate genetic improvement of these traits and the breeding of elite crop varieties. For example, a common wild rice species, *Oryza rufipogon*, that is considered to be the progenitor of cultivated rice (*O. sativa*), usually exhibits a prostrate growth habit, and seed shattering. Artificial selection for special alleles of *PROG1* (*PROSTRATE GROWTH 1*), *GIF1* (*GRAIN INCOMPLETE FILLING 1*), *Sh3*/*sh4* during domestication resulted in the erect growth habit, non-shattering phenotype, increased grain number, and improved grain production of modern rice cultivars (Li et al., 2006; Tan et al., 2008; Wang et al., 2008). Another cereal crop, maize (*Zea mays*), was derived from its wild ancestor *teosinte* (*Z. mays* ssp. *parviglumis*) that is highly branched and low-yielding, and special selection for overexpression of the *teosinte branched1* (*tb1*) led to the increased apical dominance found in all modern cultivars (Dong et al., 2017). In addition, a *phosphatase 2C-1* (*PP2C-1*) allele from wild soybean contributes to the increased seed weight/size in cultivated soybean accessions (Lu et al., 2017).

Cultivated peanut is an important cash crop, and is one of the main ways to obtain economic benefits for farmers in many developing countries and regions. Seeds are the most important harvest trait because seed size/weight directly determines peanut yield, and selection for large seeds has been an important target during peanut domestication and production. During the evolution of peanut, the allotetraploid wild species *A. monticola*, considered to be either the direct progenitor of the cultivated peanut or a “bridge” between the cultivated peanut and the diploid wild species, emerged much earlier and bears smaller seeds than modern cultivated peanuts with large seeds. However, the molecular basis underpinning this key transition from small to large seeds remains unknown.

### Generation and Comprehensive Analyses of Global Transcription Profiles of Developing Seeds From Both Wild and Cultivated Peanut Accessions

In the present study, we obtained transcription profiles from developing seeds for the allotetraploid wild peanut *A. monticola*, which has not been previously reported. Comprehensive transcriptome analyses were also performed between two types of peanut accessions (allotetraploid wild species and cultivated peanuts) with contrasting seed size/weight. A number of genes were differentially expressed in developing peanut seeds between the three peanut accessions; however, nearly half showed moderate expression levels (2 ≤ FPKM < 10) and only a small fraction of transcripts (1.7%–4.5%) were expressed at relatively high levels (50 ≤ FPKM). A similar phenomenon has been also observed in other cultivated peanuts (Chen X. et al., 2013, 2016; Sinha et al., 2020), and could be common among wild and cultivated peanut accessions. Some GO terms were shared among the seed developmental stages, including ‘carboxylic/organic acid biosynthetic process,’ ‘thiazole biosynthetic process,’ ‘protein synthesis and modification,’ and ‘small molecules metabolism process (thiamine biosynthetic process and flavonoid metabolic process),’ and the genes related to these terms were always up-regulated in all three peanut accessions, suggesting that the shared DEGs may be vital for increased seed size during the development of peanut pods.

Numbers of DEGs were detected in the two cultivated peanut accessions across the four developmental stages compared with the wild peanut, and the DEGs were enriched in various terms including ‘DNA replication,’ ‘modification and cell division,’ ‘carbohydrate biosynthetic/metabolic process,’ ‘photosynthesis,’ ‘protein biosynthesis, folding and deneddylation,’ ‘maintenance of seed dormancy,’ ‘phytohormone metabolic and biosynthetic process,’ and ‘cellular response to nutrient levels,’ which could imply that substantial differences exist in the seed development process between cultivated and wild peanut. Plant hormones are closely related to peanut pod and seed development (Zhu et al., 2014; Wan et al., 2017; Kumar et al., 2019; Sinha et al., 2020). Coincidentally, a number of genes related to plant hormone metabolic and biosynthetic processes, such as auxin metabolism, were identified at the early and middle stages of seed development and were found to have elevated expression levels in the cultivated peanut accessions. Gibberellin biosynthetic process-related genes showed lower expression levels during this phase, which confirmed that both auxin and GAs are important factors in the regulation of peanut seed development, and also suggested that variation in the levels of phytohormone metabolic and biosynthetic process related-transcripts may affect variations in seed phenotypes between wild and cultivated peanut accessions. Photosynthesis-related genes are more active in the aerial peg and show decreased expression in the subterranean young pod (Zhu et al., 2014; Clevenger et al., 2016; Sinha et al., 2020). Interestingly, we found that some genes involved in photosynthesis showed elevated transcript abundance at early stages of seed development in both cultivated peanut accessions. Thus, we can ask “how do these genes affect the young seed phenotype”? Little is known about the roles of photosynthesis-related genes in seed development, and more in-depth investigation needs to be done to answer this question.

### Identification of Potential Candidate Genes Associated With Peanut Seed Size/Weight Variation by Combining RNA-Seq With Mapped QTLs From Previous Reports

By using integrated analysis of GO and KEGG enrichment analyses, sequence variation, and differential gene expression, we identified three candidates (EVM0023227, EVM0031048, and EVM0062133) associated with seed size variations in the various peanut accessions. EVM0023227, which encodes topless-related protein 2, is involved in ‘cytokinesis by cell plate formation’ (GO:0000911) in the main ‘biological process’ GO category. EVM0062133, a gene that encodes transcription factor TGA7, is enriched in the ‘plant hormone signal transduction pathway.’ Although genes related to cell division and plant hormones could influence seed size/weight in plants, neither types of genes have been yet reported in peanut. Seed size is always coordinately regulated by grain filling, cell number, and cell size and shape in plants (Chun et al., 2020). Enhanced cell division can give rise to bigger rice seeds by increasing cell number (Guo et al., 2018). Interestingly, we found that some GO terms related to ‘cell division’ such as ‘nucleosome assembly,’ ‘microtubule-based process,’ ‘cytokinesis,’ and so on, were only enriched in some down-regulated genes in *A. monticola.* Majority of these GO terms related genes including EVM0023227 displayed gradually decreased expression levels during seed development for the three peanut accessions, however, a sharp decline in transcript abundance arose much later in both Line 8106 and Line 8107 (Supplementary Figures 1, 2). Whether the topless-related protein 2 affects peanut seed size through cell division remains unknown, and further cytological observations need to be performed in the future. TGA transcription factors are members of the bZIP TF family that play crucial roles in plant defense and growth (Jakoby et al., 2002); the involvement of TGA TFs in seed size and development has also not yet been described in plants. Indole-3-acetic acid (IAA) is the major auxin that influences various aspects of plant growth and development as well as responses to abiotic and biotic stresses in plant species (Ludwig-Müller, 2011). IAA conjugation is one of the mechanisms maintaining auxin homeostasis, in which conjugated compounds could serve as pool of inactive IAA and can be converted to free IAA through de-conjugation reactions modulated by a specific group of amidohydrolases (Staswick et al., 2005; Bitto et al., 2009). Coincidentally, EVM0031048, a gene that encodes IAA-amino acid hydrolase ILR1-like 5, was found to have varied nucleotide sequence, and showed relatively higher expression levels in both of the cultivated peanut accessions. Therefore, it appears that the gene EVM0031048 may contribute to variation in seed size/weight between the two cultivated peanut accessions (Lines 8106 and 8107) and the wild *A. monticola*.

A considerable amount of effort has been expended on linkage mapping and analysis of peanut seed and/or pod traits, leading to the identification of many QTLs associated with the seed size/weight phenotype and prediction of a few candidate genes for the target traits (Huang et al., 2015; Chen W. et al., 2016; Luo et al., 2017a, 2018; Lu et al., 2018; Wang Z. et al., 2018; Zhang et al., 2019; Gangurde et al., 2020). However, the causal candidate genes have yet to be isolated and characterized. Based on combined analyses of repeatedly mapped QTLs for seed size/weight on linkage group A05 from previous reports and RNA-seq data generated in the present study, several potential candidate genes were isolated successfully, of which both EVM0025654 and EVM0059693 encode putative pentatricopeptide repeat-containing proteins. Pentatricopeptide repeat (PPR) proteins are a large family of plant proteins that are characterized by the presence of degenerate 35 amino acid PPR motifs repeated in tandem. PPR proteins are involved in several aspects of gene expression both in organelle genomes and organelle biogenesis including transcription, RNA processing, splicing, stability, editing, and translation (Manna, 2015). Impairment of PPR protein function can generate abnormal phenotypes associated with embryo development, photosynthetic ability, and seed pigmentation. For example, *qKW9*, a gene that encodes a PPR, regulates maize kernel size and weight by affecting photosynthesis and grain filling (Huang et al., 2020). Recently, using linkage and association analyses, Gangurde et al. (2020) discovered several candidate genes for seed weight in peanut, one of which was a gene that is predicted to encode a PPR protein. Therefore, the PPR-containing protein-encoding gene EVM0025654 identified in this study could be a potential candidate gene associated with variation in seed size/weight in peanut.

Additionally, some important GO terms, such as ‘G2 phase of mitotic interphase,’ ‘nucleosome assembly,’ ‘DNA-dependent DNA replication and DNA modification,’ and cytokinin-activated signaling pathway’ were also significantly enriched, and the related genes displayed differential expression levels between the wild and cultivated peanut accessions. The majority of these showed no nucleotide variations in their genomic sequences. A partial explanation for this is that these genes may be part of some molecular networks, and their expression levels could be influenced by downstream or upstream factors. Alternatively, these DEGs may be related to the differential methylation levels that exist between wild *A. monticola* and cultivated peanut accessions during early seed development (Wang P. et al., 2018), although this needs further verification.

In conclusion, we generated transcriptome libraries from developing seeds of two cultivated peanut accessions and wild *A. monticola* at 15, 30, 45, and 60 DPF, and also performed comprehensive transcriptomic analyses on the libraries. Also, through a combined method using DGEA (differential gene expression analysis), GO term and KEGG pathway enrichment, sequence variation analysis, and mapping QTLs from previous reports, we identified several potential candidate genes for variation in seed size/weight between the tetraploid wild *A. monticola* and the two cultivated peanut accessions. By genotyping both peanut landraces and genetic segregated population, EVM0025654 encoding a PPR protein has been shown to be possibly associated with the increased seed size/weight of the cultivated accessions in comparison with the allotetraploid wild peanut. The data generated here will be an invaluable resource for the genetic dissection of peanut domestication-related traits, and our results provide additional insights into the identification and functions of causal candidate genes responsible for the variation in seed size/weight in peanut.

## Data Availability Statement

All raw sequences for transcriptome are available in the NCBI Sequence Read Archive under accession numbers: SRR8334380, SRR8334357, SRR8334346, and SRR8334385 for *A. monticola* 15–60 DPF; SRR8334375–SRR8334377 and SRR8334345 for Line 8107 15–60 DPF; SRR8334371–SRR8334374 for Line 8106 15–60 DPF, respectively.

## Author Contributions

DY conceived, supervised the experiment, and revised the manuscript. ZL analyzed the data and wrote the draft manuscript. ZL and KKZ performed research. XZ, XM, and FG assisted in editing the manuscript. KZ, CQ, and GG managed field research and plant propagation. All authors have read and approved the final manuscript.

## Conflict of Interest

The authors declare that the research was conducted in the absence of any commercial or financial relationships that could be construed as a potential conflict of interest.

## Footnotes

1 http://www.fao.org/faostat/zh/#data/QC

2 http://bioinfo.ut.ee/primer3-0.4.0/

## Abbreviations

DEG: differentially expressed gene
DPF: days past flowering
FPKM: fragments per kilobase of transcript length per million mapped reads
GO: Gene Ontology
KEGG: Kyoto Encyclopedia of Genes and Genomes
PPR: pentatricopeptide repeat protein
QTL: quantitative trait locus
RNA-seq: RNA sequencing
SNP: single nucleotide polymorphism.
