# Supplementary material for: Comprehensive Transcriptome Analyses Reveal Candidate Genes for Variation in Seed Size/Weight During Peanut (Arachis hypogaea L.) Domestication
Source: Front Plant Sci. 2021 May 19;12:666483. doi: 10.3389/fpls.2021.666483 (PMC8170302; doi:10.3389/fpls.2021.666483)
Supplement: Supplementary file 1 [file Data_Sheet_1.PDF]

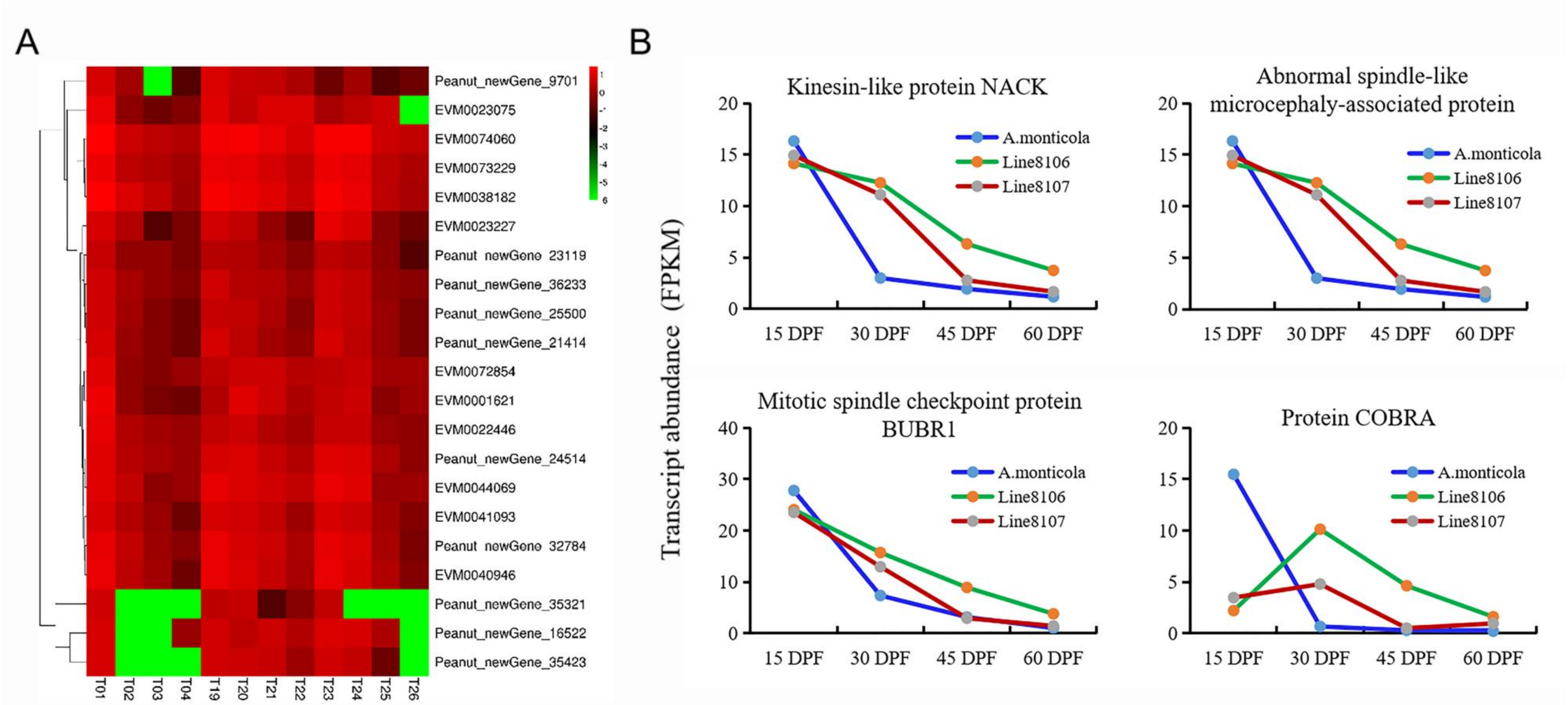

Figure S1. Heatmap and representatives of some DEGs shared by the GO terms 'microtubule cytoskeleton organization', 'cytokinesis', and 'mitotic G2 phase' in *A. monticola.* **(A)** Differentially expressed genes (DEGs) shared among the 30, 45, and 60 DPF samples from *A. monticola* were used to perform GO enrichment analysis, and 21 of these DEGs were used to construct the heatmap. **(B)** Expression of some representatives of the 21 DEGs is shown.
