# Supplementary material for: Comprehensive Transcriptome Analyses Reveal Candidate Genes for Variation in Seed Size/Weight During Peanut (Arachis hypogaea L.) Domestication
Source: Front Plant Sci. 2021 May 19;12:666483. doi: 10.3389/fpls.2021.666483 (PMC8170302; doi:10.3389/fpls.2021.666483)
Supplement: Supplementary file 2 [file Data_Sheet_2.PDF]

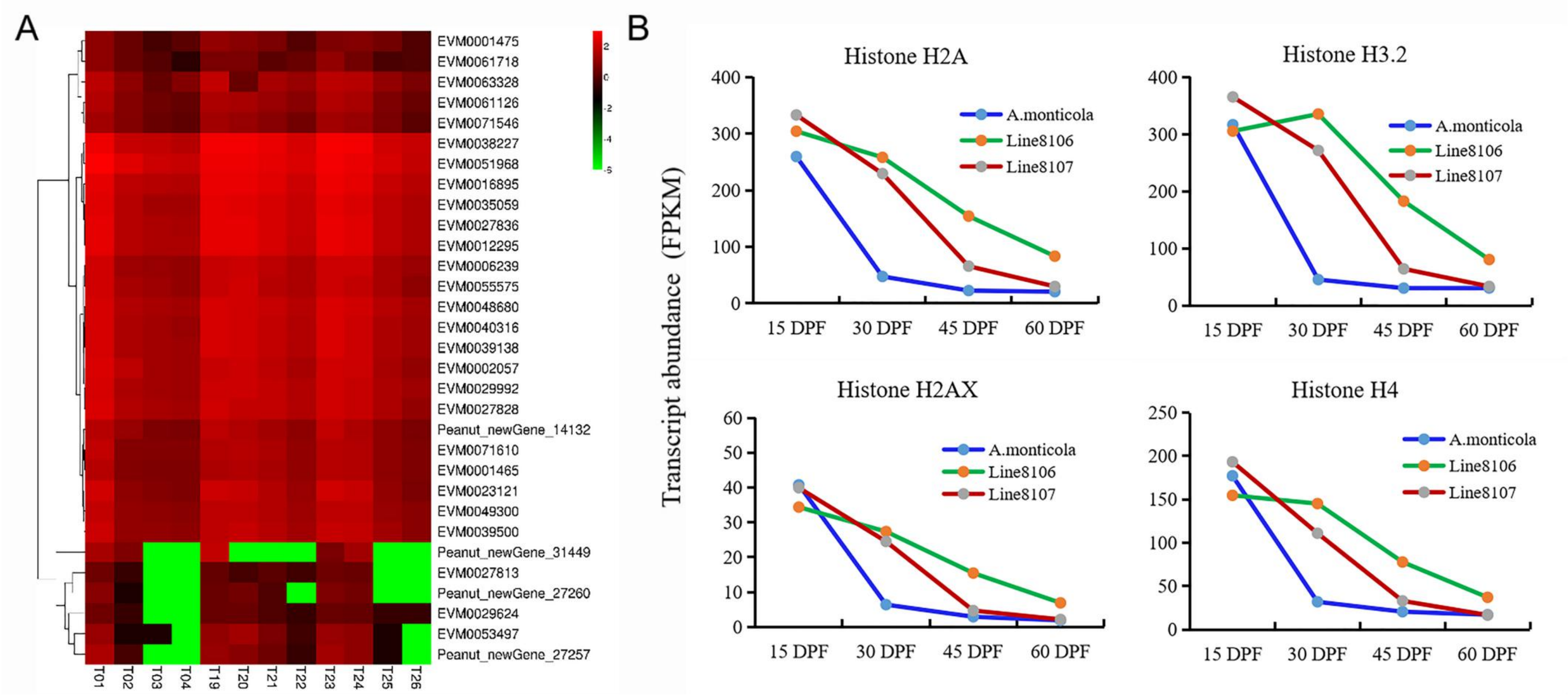

Figure S2. Heatmap and representatives of the GO term ‘nucleosome assembly’ enrichment-related DEGs in *A. monticola.*

(A) DEGs shared among the 30, 45, and 60 DPF samples from *A. monticola* were used to perform GO enrichment analysis, and 31 DEGs were used to construct the heatmap. **(B)** Expression of some representatives of the 31 DEGs.
