# Supplementary material for: Comprehensive Transcriptome Analyses Reveal Candidate Genes for Variation in Seed Size/Weight During Peanut (Arachis hypogaea L.) Domestication
Source: Front Plant Sci. 2021 May 19;12:666483. doi: 10.3389/fpls.2021.666483 (PMC8170302; doi:10.3389/fpls.2021.666483)
Supplement: Supplementary file 3 [file Data_Sheet_3.pdf]

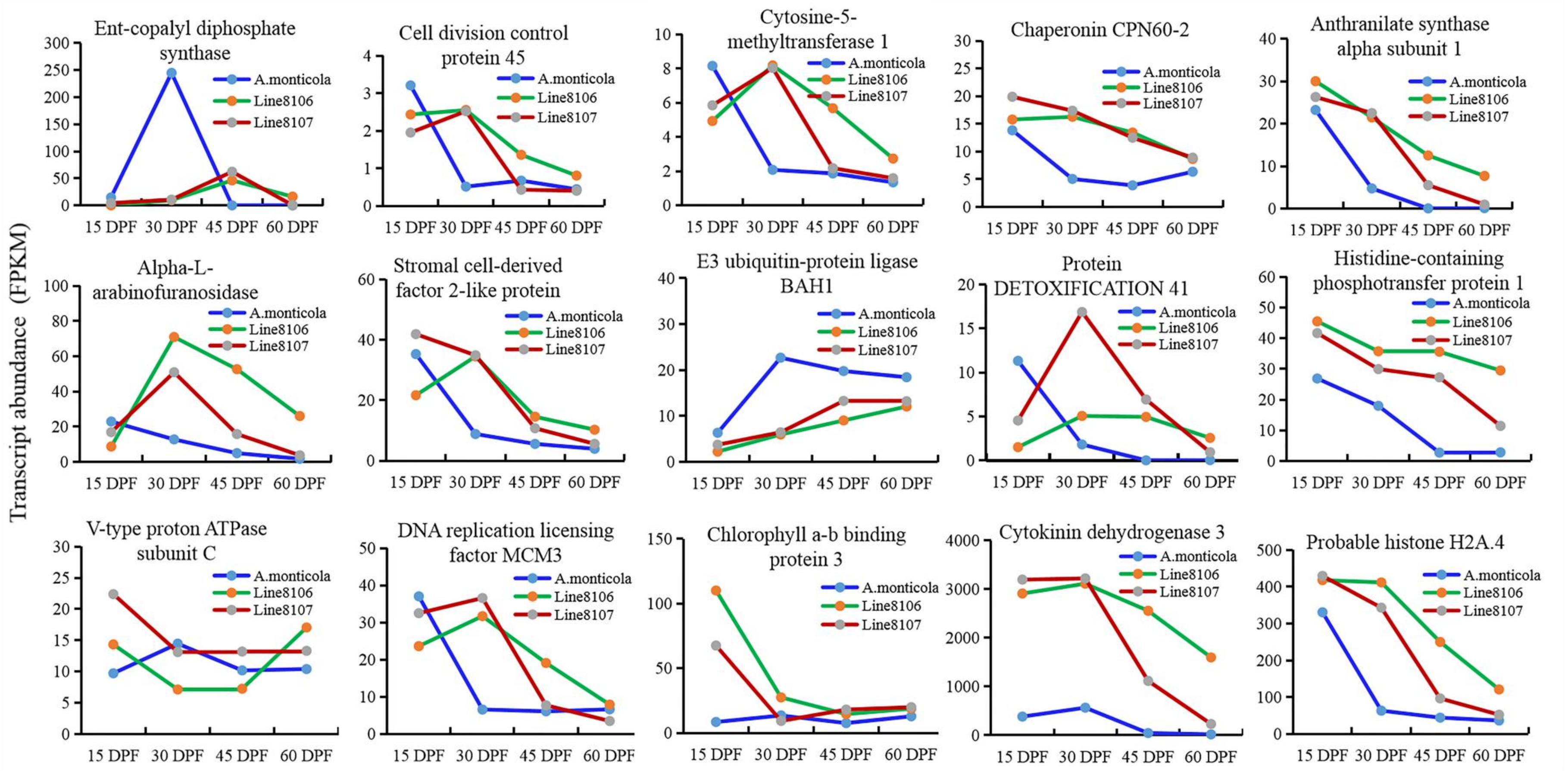

Figure S3. Representatives of some significantly enriched GO term-related DEGs between the wild *A. monticola* and cultivated peanut Lines 8107/8106. GO enrichment analysis was performed based on the DEGs between the wild and cultivated peanut accessions at 15, 30, 45, and/or 60 DPF (**Figure 4C-D**). For some of the significantly enriched GO terms, single representative genes were randomly selected and the corresponding transcript abundance was determined.
