# Supplementary material for: Comprehensive Transcriptome Analyses Reveal Candidate Genes for Variation in Seed Size/Weight During Peanut (Arachis hypogaea L.) Domestication
Source: Front Plant Sci. 2021 May 19;12:666483. doi: 10.3389/fpls.2021.666483 (PMC8170302; doi:10.3389/fpls.2021.666483)
Supplement: Supplementary file 4 [file Data_Sheet_4.doc]

**Supplementary tables (Table S1-11)**

**Table S1. Summary of some peanut accessions for analyzing genomic sequence variations**

| Accession ID | 100-seed weight (g) | Origin |
| --- | --- | --- |
| Tifrunner (PI644011) | 40.0 | The U.S.A. |
| US-3 | 45.3 | The U.S.A. |
| Q2 | 95.4 | China |
| Q3 | 71.6 | China |
| Q4 | 79.4 | China |
| Q5 | 69.0 | China |
| Q6 | 39.0 | China |
| Q7 | 85.1 | China |
| Q8 | 51.8 | China |
| Q10 | 56.4 | China |
| Q12 | 109.7 | China |
| Q14 | 55.2 | China |
| Q15 | 118.7 | China |
| Q16 | 81.9 | China |
| Q17 | 69.6 | China |
| Z88 | 77.5 | China |
| Z94 | 75.0 | China |
| Zp06 | 106.2 | China |
| Yuanza9102 | 70.5 | China |
| D7500 | 28.0 | China |
| Zp08 | 97.0 | China |
| Hua103 | 74.0 | China |
| Hua2014 | 67.9 | China |
| Hua814 | 44.0 | China |
| Shitouqi | 52.5 | China |
| Fuhuasheng | 74.0 | China |
| Haihua1 | 90.0 | China |
| Line8106 | 100.6 | China |
| Line8107 | 144.0 | China |

**Table S2.** Functional annotation of expressed genes in three peanut accessions by using several databases

| Materials | COG | GO | KEGG | Swiss-Prot | eggNOG | Nr | Number of total annotated genes (% *) |
| --- | --- | --- | --- | --- | --- | --- | --- |
| *A. monticola* | 12436 | 18155 | 13176 | 23903 | 25299 | 27428 | 35066 (95.31) |
| Line 8106 | 12603 | 18380 | 13222 | 24181 | 25620 | 27753 | 35378 (95.01) |
| Line 8107 | 12622 | 18421 | 13219 | 24234 | 25785 | 27732 | 35511 (94.99) |

* The number of total annotated genes was divided by that of totally expressed genes ×100 for each of *A.monticola*, Line 8106, and Line 8107, respectively

**Table S3.** Known, new and totally expressed genes identified in 12 seed samples from Line 8106, Line 8107 and *A.monticola*

| Type | T01 | T02 | T03 | T04 | T19 | T20 | T21 | T22 | T23 | T24 | T25 | T26 |
| --- | --- | --- | --- | --- | --- | --- | --- | --- | --- | --- | --- | --- |
| Number of expressed new gene | 5650 | 5604 | 5310 | 5314 | 5283 | 5295 | 5290 | 5194 | 5320 | 5392 | 5253 | 5078 |
| Number of predicted genes in the reference genome of *A. monticola* * | 30867 | 30853 | 30244 | 30105 | 30337 | 30345 | 30475 | 30302 | 30486 | 30533 | 30421 | 30073 |
| Number of total expressed genes | 36517 | 36457 | 35554 | 35419 | 35620 | 35640 | 35765 | 35496 | 35806 | 35925 | 35674 | 35151 |

T1-T4, T19-T22, and T23-T26 indicate the seed samples at 15, 30, 45, and 60 DPF from *A. monticola* (*A. mon*), Line 8106, and Line 8107, respectively. * The reference assembly of *A. monticola* was reported by Yin et al. (2018) and used in the present study

**Table S4.** Significantly enriched GO terms based on DEGs shared among three seed developmental stages (30, 45, and 60 DPF) within each of three peanut accessions

| DEGs used for GO enrichment analyses | Significant GO terms based on T1-4  shared DEGs ^a^ | Significant GO terms based on T23-26 shared DEGs ^a^ | Significant GO terms based on T19-22  shared DEGs ^a^ |
| --- | --- | --- | --- |
| Up-regulated DEGs ^b^ | Photosynthesis-related: photosynthesis, light harvesting (GO:0009765); regulation of photosynthesis  (GO:0042548) | Thiazole related: thiazole biosynthetic process (GO:0052837); | Thiazole related: thiazole biosynthetic process(GO:0052837); |
|  | Thiazole biosynthetic/metabolic process:oxazole or thiazole biosynthetic process(GO:0018131);thiazole biosynthetic process(GO:0052837);thiazole metabolic process(GO:0052838);oxazole metabolic process(GO:0046484) | Protein transport,location & biosynthesis related:protein targeting to peroxisome(GO:0006625);protein localization to peroxisome(GO:0072662);protein import into peroxisome matrix(GO:0016558);cellular amino acid biosynthetic process(GO:0008652) | Carboxylic/organic acid related: carboxylic acid biosynthetic process(GO:0046394);monocarboxylic acid metabolic process(GO:0032787);monocarboxylic acid biosynthetic process(GO:0072330); |
|  | Carboxylic/organic acid related:carboxylic acid biosynthetic process(GO:0046394);organic acid biosynthetic process(GO:0016053);monocarboxylic acid biosynthetic process(GO:0072330);tricarboxylic acid metabolic process(GO:0072350) | Carboxylic/organic acid related: organic acid biosynthetic process(GO:0016053);carboxylic acid biosynthetic process(GO:0046394);tricarboxylic acid metabolic process(GO:0072350) | Fatty acid related:fatty acid metabolic process(GO:0006631);fatty acid biosynthetic process(GO:0006633); |
|  | Protein related:organonitrogen compound biosynthetic process(GO:1901566);negative regulation of peptidase activity(GO:0052547);protein folding(GO:0006457);protein-chromophore linkage(GO:0018298);protein autophosphorylation(GO:0046777) | Small molecule & thiamine related:small molecule biosynthetic process(GO:0044283);thiamine biosynthetic process(GO:0009228); | Protein related:organonitrogen compound biosynthetic process(GO:1901566);cellular amino acid biosynthetic process(GO:0008652);alpha-amino acid biosynthetic process(GO:1901607);protein autophosphorylation(GO:0046777);protein folding(GO:0006457);glycine biosynthetic process(GO:0006545); |
|  | Small molecule & flavonoid related: flavonoid metabolic process(GO:0009812);small molecule biosynthetic process(GO:0044283) | Cell cycle related: mitotic G2 phase (GO:0000085); mitotic interphase (GO:0051329); interphase (GO:0051325); biological phase (GO:0044848) | Stress-related:response to nutrient levels(GO:0031667);response to molecule of fungal origin(GO:0002238);response to toxic substance(GO:0009636);cellular response to nitrogen starvation(GO:0006995) |
|  | Nucleoside biosynthesis related:purine ribonucleoside biosynthetic process(GO:0046129);ribonucleoside biosynthetic process(GO:0042455) |  | Small molecule,flavonoid & thiamine related:flavonoid metabolic process(GO:0009812);thiamine biosynthetic process(GO:0009228);proanthocyanidin biosynthetic process(GO:0010023) |
|  | Sugar-related:aminoglycan metabolic process(GO:0006022);aminoglycan catabolic process(GO:0006026);amino sugar catabolic process(GO:0046348) |  | Sugar-related:xylan catabolic process(GO:0045493);hexose phosphate transport(GO:0015712);triose phosphate transmembrane transport(GO:0035436) |
| Down-regulated DEGs ^c^ | Cell division related:nucleosome assembly(GO:0006334);microtubule-based process(GO:0007017);cytokinesis(GO:0000910):microtubule cytoskeleton organization(GO:0000226);mitotic chromosome condensation(GO:0007076);mitotic G2 phase(GO:0000085);cell fate commitment(GO:0045165) | Amino acid biosynthesis-related: organonitrogen compound biosynthetic process(GO:1901566);cellular amino acid biosynthetic process(GO:0008652);aromatic amino acid family biosynthetic process(GO:0009073) | Pigment related: heme biosynthetic process(GO:0006783);heme metabolic process(GO:0042168);porphyrin-containing compound biosynthesis(GO:0006779);photosynthesis, light harvesting(GO:0009765); |
|  | Seed dormancy related:maintenance of dormancy(GO:0097437);maintenance of seed dormancy(GO:0010231) | Pigment related:pigment biosynthetic process(GO:0046148);pigment metabolic process(GO:0042440);heme biosynthetic process(GO:0006783);heme metabolic process(GO:0042168) | Stress-related:cellular response to hydrogen peroxide(GO:0070301);response to nutrient levels(GO:0031667) |
|  | Pigment related: pigment metabolic process(GO:0042440);proanthocyanidin biosynthetic process(GO:0010023) | Stress-related: response to nutrient levels(GO:0031667);response to high light intensity(GO:0009644) | Cell development related:regulation of cell morphogenesis involved(GO:0010769);regulation of cell development(GO:0060284) |
| Up-/down-regulated DEGs | Gibberellin biosynthetic process(GO:0009686) |  | Gibberellin metabolic process(GO:0009685) |

^a^ Compared with 15 DPF, the common DEGs among at 30, 45, and 60 DPF within each of three peanut accessions were designated T1-4, T19-22 and T23-26 shared DEGs, respectively. ^b^ and ^c^ represented the genes showing elevated or reduced expression levels across 30 to 60 DPF relative to 15 DPF in *A. monticola*, Line 8106 and Line 8107, respectively. DEG, differentially-expressed gene; DPF, days past flowering

**Table S5.** Statistic of gene sets with differential expression within the three peanut accessions

| Materials | DEGs set | Number of up-regulated genes | Number of down-regulated genes | Number of total DEGs |
| --- | --- | --- | --- | --- |
| 1. *monticola* | 15 DPF vs. 30 DPF | 3529 | 3691 | 12615 |
|  | 15 DPF vs. 45 DPF | 3703 | 4572 |  |
|  | 15 DPF vs. 60 DPF | 3388 | 4902 |  |
| Line 8106 | 15 DPF vs. 30 DPF | 3009 | 3162 | 11843 |
|  | 15 DPF vs. 45 DPF | 3873 | 3299 |  |
|  | 15 DPF vs. 60 DPF | 3966 | 4051 |  |
| Line 8107 | 15 DPF vs. 30 DPF | 2253 | 2427 | 12208 |
|  | 15 DPF vs. 45 DPF | 3879 | 3746 |  |
|  | 15 DPF vs. 60 DPF | 3590 | 4343 |  |

DEG, differentially-expressed gene; DPF, days past flowering. 15, 30, 45 and 60 DPF indicate the seed samples at 15, 30, 45, and 60 days past flowering (DPF) from *A. monticola,* Line 8107, Line 8106 respectively

**Table S6.** Some QTLs for the seed size/weight phenotype on linkage group A05 detected repeatably from previous studies

| Traits | QTLs | Mapping population | Reference genome | Flanking marker for mapping  interval | Genetic/ Physical length | PVE (%) | Liturature |
| --- | --- | --- | --- | --- | --- | --- | --- |
| PL | qPLA05.7 | RIL | *A. duranensis* | A05A1430-A05A1601 | 3.7 cM/ 2.47 Mb | 13.75-26.82 | Luo et al. 2017 |
| PW | *qHPWA05.6* |  |  | A05A1430-A05A1601 |  | 16.89-27.84 |  |
| HPW | *qHPWA05.2* | RIL | *A. duranensis* | Ad05A20262-AHGA160418 | 1.3 cM/280 kb | 10.38-14.66 | Luo et al. 2018 |
| PL | *qPLA05.1* |  |  |  |  | 11.5-16.44 |  |
| PW | *qPWA05.2* |  |  |  |  | 6.99-8.38 |  |
| Pod weight | *MQTL_100PW_A05.5* | * | *Arachis hypogaea* L. | A05A1471-A05A1562 | 0.7 cM/630kb | 19-31 | Lu et al. 2018* |
| Pod size | *MQTL_PL_A05.5* | * |  |  |  |  |  |
| Pod size | *MOTL_PW_PA05.3* | * |  |  |  |  |  |
| Pod weight | *qPW_A05-2* | NAM_Tifrunner | *Arachis hypogaea* L. | Affx-152072578-Affx-152034828 | 1.2 cM/- | 33.30 | Gangurde et al. 2019 |
| Seed weight | *qSW_A05-1* |  |  |  |  | 30.60 |  |
| Pod weight | qPW_A05-2 | NAM_Florida-07 | *Arachis hypogaea* L. | Affx-152026889-Affx-152055218 | 14 cM/- | 21.80 | Gangurde et al. 2019 |
| Seed weight | *qSW_A05-3* |  |  |  |  | 40.30 |  |

RIL, recombinant inbred line mapping population; MQTLs, meta-QTLs; PL, pod length; PW, pod width; HPW, hundred-pod weight; NAM, nested-association mapping population.

* Meta-analysis based on other reports was performed by Lu et al. (2018). PVE, phenotypic variation explained; The genome assembly of peanut accession V14167A and Tifrunner was used, respectively

**Table S7.** SNP variations in the integrated mapping intervals on chromosome A.mon-A05 of cultivated peanut Line 8106 and Line 8107

| Locus | Gene_ID | ORF on +/- strand | Physical  position (bp) | Reference ^a^ | Variation ^b^ | SNP effect | Codon change | Predicted gene function ^e^ |
| --- | --- | --- | --- | --- | --- | --- | --- | --- |
| *Region1* | EVM0025146 | + | 116463421 | C | T /Line 8107 ^c^ | Non-synonymous | aCc →aTc | Protein farnesyltransferase subunit beta |
|  | EVM0074021 | - | 119625225 | A | T | UTR_3_prime | . | Uncharacterized protein |
| *Region2* | EVM0024505 | - | 109483454 | T | A /Line 8107 ^c^ | Non-synonymous | tAt →tTt | Serine/threonine-protein kinase |
|  | EVM0027962 | - | 109865939 | T | C | Non-synonymous | gAt →gGt | Uncharacterized protein |
|  | EVM0046635 | + | 111137051 | A | T /Line 8107 ^c^ | Synonymous | gcA →gcT | Serine/threonine-protein kinase EDR1 |
|  | EVM0025654 | - | 111201604 | C | G | Non-synonymous | tGt →tCt | Pentatricopeptide repeat-containing protein |
|  | EVM0058772 | + | 111472535 | G | T /Line 8106 ^d^ | Non-synonymous | aGt →aTt | Pentatricopeptide repeat-containing protein |
|  | EVM0059693 | + | 111487529 | G | C | Non-synonymous | gGg →gCg | Pentatricopeptide repeat-containing protein |
|  |  |  | 111490408 | A | T /Line 8106 ^d^ | UTR_3_PRIME | . |  |
|  | EVM0029388 | + | 112749715 | A | C /Line 8107 ^c^ | Synonymous | acA →acC | Pentatricopeptide repeat-containing protein |
|  | EVM0029171 | - | 114785708 | G | A /Line 8106 ^d^ | Non-synonymous | Cgt →Tgt | Pentatricopeptide repeat-containing protein |

^a^ indicate the genotypes of the SNP loci in the “+” strand of the *A.monticola* reference genome while ^b^ represents the genotypes of the corresponding locus present in the genomes of cultivated peanut accessions Line 8106 and Line 8107. ^c^ and ^d^ refer to the special genotype at the SNP locus only existing in the genome of Line 8107 or Line 8106, respectively. ^e^ The gene function was predicted according to the Swiss_Prot and Nr database. ORF, open reading frame

**Table S8.** SNP marker development for sequence variation test in the identified candidate genes associated with peanut seed size/weight phenotype

| Marker name | Sequence (5' to 3') | | Product  size (bp) | Target gene | SNP variation |
| --- | --- | --- | --- | --- | --- |
| SNP048 | Forward primer sequence: | GAGCCACTTTTCTTGCGACG | 902 | EVM0031048 | C>T |
|  | Reverse primer sequence: | AGGCATGACAAATCAAAGGGT |  |  |  |
| SNP021 | Forward primer sequence: | CCAGCCCTGTTCAAGCAAAC | 837 | EVM0074021 | A>T |
|  | Reverse primer sequence: | CATCCCCTGTCTCAACGCTT |  |  |  |
| SNP133 | Forward primer sequence: | TCCGGAAGGATCCCCTGATT | 826 | EVM0062133 | A>C |
|  | Reverse primer sequence: | TGTGGGGTTTCTTATGGAGCA |  |  |  |
| SNP227 | Forward primer sequence: | TGGCTGGATTGGTATTCGCT | 982 | EVM0023227 | T>A |
|  | Reverse primer sequence: | TCGAGTTTAAAGCTCAAACCCTAAC |  |  |  |
| SNP654 | Forward primer sequence: | AGCCCATCAATCAAGGTGGT | 687 | EVM0025654 | C>G |
|  | Reverse primer sequence: | TGGTTCTTTGCTTTGGTGTCA |  |  |  |
| SNP962 | Forward primer sequence: | GCTTGGACTGAACTCGGGAA | 879 | EVM0027962 | T>C |
|  | Reverse primer sequence: | GAATCGAGGAACTCCCGAACA |  |  |  |

**Table S9.** Enriched GO terms based on stage-specific genes expressed in *A. monticola*

| Stage | GO.ID | Term | Significant ^a^ | KS ^b^ |
| --- | --- | --- | --- | --- |
| 15 DPF | GO:0006909 | phagocytosis | 2 | 0.0033 |
|  | GO:0010388 | cullin deneddylation | 4 | 0.004 |
|  | GO:0000085 | mitotic G2 phase | 4 | 0.004 |
|  | GO:0006535 | cysteine biosynthetic process from serine | 2 | 0.0093 |
|  | GO:1901566 | organonitrogen compound biosynthetic process | 44 | 0.013 |
|  | GO:0072522 | purine-containing compound biosynthetic process | 5 | 0.0206 |
|  | GO:0006783 | heme biosynthetic process | 4 | 0.0232 |
|  | GO:0007166 | cell surface receptor signaling pathway | 5 | 0.027 |
|  | GO:0042493 | response to drug | 14 | 0.0317 |
|  | GO:0006855 | drug transmembrane transport | 14 | 0.0317 |
|  | GO:0015893 | drug transport | 14 | 0.0317 |
|  | GO:0007186 | G-protein coupled receptor signaling pathway | 3 | 0.0318 |
|  | GO:0045493 | xylan catabolic process | 4 | 0.0322 |
|  | GO:0042168 | heme metabolic process | 4 | 0.0364 |
|  | GO:0055085 | transmembrane transport | 56 | 0.0381 |
|  | GO:0006457 | protein folding | 6 | 0.0388 |
|  | GO:0006547 | histidine metabolic process | 2 | 0.0395 |
|  | GO:0052803 | imidazole-containing compound metabolic process | 2 | 0.0395 |
|  | GO:0010417 | glucuronoxylan biosynthetic process | 2 | 0.0418 |
|  | GO:0009812 | flavonoid metabolic process | 5 | 0.0419 |
|  | GO:0006779 | porphyrin-containing compound biosynthetic process | 6 | 0.0437 |
|  | GO:0072350 | tricarboxylic acid metabolic process | 2 | 0.0447 |
|  | GO:0009156 | ribonucleoside monophosphate biosynthetic process | 6 | 0.0464 |
|  | GO:0010496 | intercellular transport | 3 | 0.0472 |
|  | GO:0019932 | second-messenger-mediated signaling | 3 | 0.0483 |
|  | GO:0006261 | DNA-dependent DNA replication | 20 | 0.0499 |
| 30 DPF | GO:0006535 | cysteine biosynthetic process from serine | 2 | 0.0141 |
|  | GO:0055081 | anion homeostasis | 2 | 0.0151 |
|  | GO:0031425 | chloroplast RNA processing | 2 | 0.0273 |
|  | GO:0006779 | porphyrin-containing compound biosynthetic process | 2 | 0.0439 |
|  | GO:0009853 | photorespiration | 3 | 0.0384 |
|  | GO:0006625 | protein targeting to peroxisome | 3 | 0.0417 |
|  | GO:0072662 | protein localization to peroxisome | 3 | 0.0417 |
|  | GO:0072663 | establishment of protein localization to peroxisome | 3 | 0.0417 |
|  | GO:0043574 | peroxisomal transport | 3 | 0.0417 |
|  | GO:0072522 | purine-containing compound biosynthetic process | 4 | 0.0096 |
|  | GO:0006891 | intra-Golgi vesicle-mediated transport | 4 | 0.0369 |
|  | GO:0006457 | protein folding | 5 | 0.014 |
|  | GO:0042594 | response to starvation | 5 | 0.0146 |
|  | GO:0031667 | response to nutrient levels | 5 | 0.0146 |
|  | GO:0009267 | cellular response to starvation | 5 | 0.0225 |
|  | GO:0031669 | cellular response to nutrient levels | 5 | 0.0225 |
|  | GO:0046148 | pigment biosynthetic process | 5 | 0.041 |
|  | GO:0042440 | pigment metabolic process | 5 | 0.0463 |
|  | GO:1901566 | organonitrogen compound biosynthetic process | 16 | 0.0389 |
|  | GO:0055085 | transmembrane transport | 30 | 0.0399 |
| 45 DPF | GO:1901566 | organonitrogen compound biosynthetic process | 2 | 0.0037 |
|  | GO:0055085 | transmembrane transport | 5 | 0.0258 |
|  | GO:0043094 | cellular metabolic compound salvage | 3 | 0.0521 |
| 60 DPF | GO:1901071 | glucosamine-containing compound metabolic process | 2 | 0.0044 |
|  | GO:1901566 | organonitrogen compound biosynthetic process | 4 | 0.0111 |
|  | GO:0007166 | cell surface receptor signaling pathway | 2 | 0.0278 |
|  | GO:0008652 | cellular amino acid biosynthetic process | 2 | 0.0306 |
|  | GO:0055085 | transmembrane transport | 4 | 0.0341 |

^a^ The number of the genes enriched significantly in the corresponding GO term. ^b^ The *P* value <0.05 indicates the genes are significantly enriched in some GO term. 15, 30, 45 and 60 DPF indicate the seed samples at 15, 30, 45, and 60 days past flowering (DPF) from *A. monticola*, respectively

**Table S10.** Enriched GO terms based on stage-specific genes expressed in Line 8106

| Stage | GO.ID | Term | Significant ^a^ | KS ^b^ |
| --- | --- | --- | --- | --- |
| 15 DPF | GO:0072522 | purine-containing compound biosynthetic process | 5 | 0.0054 |
|  | GO:0006457 | protein folding | 10 | 0.0084 |
|  | GO:1901566 | organonitrogen compound biosynthetic process | 18 | 0.0187 |
|  | GO:0009269 | response to desiccation | 3 | 0.0195 |
|  | GO:0055085 | transmembrane transport | 29 | 0.0205 |
|  | GO:0006557 | S-adenosylmethioninamine biosynthetic process | 2 | 0.021 |
|  | GO:0046499 | S-adenosylmethioninamine metabolic process | 2 | 0.021 |
|  | GO:0006779 | porphyrin-containing compound biosynthetic process | 4 | 0.0228 |
|  | GO:0055114 | oxidation-reduction process | 74 | 0.0297 |
|  | GO:0042401 | cellular biogenic amine biosynthetic process | 3 | 0.036 |
|  | GO:0009309 | amine biosynthetic process | 3 | 0.036 |
|  | GO:0046148 | pigment biosynthetic process | 7 | 0.0361 |
|  | GO:0042440 | pigment metabolic process | 8 | 0.0434 |
|  | GO:0018298 | protein-chromophore linkage | 2 | 0.0456 |
|  | GO:0042451 | purine nucleoside biosynthetic process | 2 | 0.0458 |
|  | GO:0046129 | purine ribonucleoside biosynthetic process | 2 | 0.0458 |
| 30 DPF | GO:1901566 | organonitrogen compound biosynthetic process | 13 | 0.0041 |
|  | GO:0022403 | cell cycle phase | 1 | 0.0136 |
|  | GO:0044848 | biological phase | 1 | 0.0136 |
|  | GO:0000085 | mitotic G2 phase | 1 | 0.0136 |
|  | GO:0051325 | interphase | 1 | 0.0136 |
|  | GO:0051329 | mitotic interphase | 1 | 0.0136 |
|  | GO:0051319 | G2 phase | 1 | 0.0136 |
|  | GO:0055085 | transmembrane transport | 11 | 0.0224 |
|  | GO:0009877 | nodulation | 2 | 0.0231 |
|  | GO:0072350 | tricarboxylic acid metabolic process | 3 | 0.0287 |
|  | GO:0008652 | cellular amino acid biosynthetic process | 5 | 0.0396 |
|  | GO:0042401 | cellular biogenic amine biosynthetic process | 2 | 0.0461 |
|  | GO:0009309 | amine biosynthetic process | 2 | 0.0461 |
|  | GO:0006779 | porphyrin-containing compound biosynthetic process | 2 | 0.0477 |
|  | GO:0080129 | proteasome core complex assembly | 2 | 0.0554 |
| 45 DPF | GO:1901566 | organonitrogen compound biosynthetic process | 4 | 0.004 |
|  | GO:0006457 | protein folding | 2 | 0.0128 |
|  | GO:0042594 | response to starvation | 2 | 0.0207 |
|  | GO:0031667 | response to nutrient levels | 2 | 0.0207 |
|  | GO:0055085 | transmembrane transport | 15 | 0.0224 |
|  | GO:0009267 | cellular response to starvation | 2 | 0.0316 |
|  | GO:0031669 | cellular response to nutrient levels | 2 | 0.0316 |
|  | GO:0008652 | cellular amino acid biosynthetic process | 3 | 0.043 |
|  | GO:0055114 | oxidation-reduction process | 35 | 0.0444 |
| 60 DPF | GO:0006457 | protein folding | 5 | 0.004 |
|  | GO:1901566 | organonitrogen compound biosynthetic process | 3 | 0.0054 |
|  | GO:0072522 | purine-containing compound biosynthetic process | 2 | 0.0136 |
|  | GO:0009644 | response to high light intensity | 3 | 0.0223 |
|  | GO:0006887 | exocytosis | 2 | 0.0241 |
|  | GO:0055085 | transmembrane transport | 19 | 0.0268 |
|  | GO:0042451 | purine nucleoside biosynthetic process | 2 | 0.0427 |
|  | GO:0046129 | purine ribonucleoside biosynthetic process | 2 | 0.0427 |
|  | GO:0006979 | response to oxidative stress | 6 | 0.0457 |
|  | GO:0010257 | NADH dehydrogenase complex assembly | 1 | 0.0482 |
|  | GO:0052548 | regulation of endopeptidase activity | 2 | 0.0488 |
|  | GO:0010951 | negative regulation of endopeptidase activity | 2 | 0.0488 |

^a^ The number of the genes enriched significantly in the corresponding GO term. ^b^ The *P* value <0.05 indicates the genes are significantly enriched in some GO term. 15, 30, 45 and 60 DPF indicate the seed samples at 15, 30, 45, and 60 days past flowering (DPF) from Line 8106, respectively

**Table S11.** Enriched GO terms based on stage-specific genes expressed in Line 8107

| Stage | GO.ID | Term | Significant ^a^ | KS ^b^ |
| --- | --- | --- | --- | --- |
| 15 DPF | GO:0006457 | protein folding | 7 | 0.0055 |
|  | GO:0006535 | cysteine biosynthetic process from serine | 2 | 0.0059 |
|  | GO:0072522 | purine-containing compound biosynthetic process | 5 | 0.0077 |
|  | GO:0031425 | chloroplast RNA processing | 4 | 0.0085 |
|  | GO:0042440 | pigment metabolic process | 11 | 0.0162 |
|  | GO:0006779 | porphyrin-containing compound biosynthetic process | 7 | 0.0217 |
|  | GO:1901566 | organonitrogen compound biosynthetic process | 31 | 0.0256 |
|  | GO:0006783 | heme biosynthetic process | 2 | 0.0306 |
|  | GO:0055085 | transmembrane transport | 31 | 0.0312 |
|  | GO:0046148 | pigment biosynthetic process | 11 | 0.0314 |
|  | GO:0072488 | ammonium transmembrane transport | 2 | 0.0319 |
|  | GO:0033014 | tetrapyrrole biosynthetic process | 8 | 0.0344 |
|  | GO:0015696 | ammonium transport | 4 | 0.0359 |
|  | GO:0042451 | purine nucleoside biosynthetic process | 3 | 0.0443 |
|  | GO:0046129 | purine ribonucleoside biosynthetic process | 3 | 0.0443 |
|  | GO:0042168 | heme metabolic process | 2 | 0.0455 |
|  | GO:0009152 | purine ribonucleotide biosynthetic process | 5 | 0.0456 |
|  | GO:0042594 | response to starvation | 3 | 0.048 |
|  | GO:0031667 | response to nutrient levels | 3 | 0.048 |
|  | GO:0042430 | indole-containing compound metabolic process | 6 | 0.0481 |
|  | GO:0009156 | ribonucleoside monophosphate biosynthetic process | 5 | 0.0499 |
| 30 DPF | GO:1901566 | organonitrogen compound biosynthetic process | 13 | 0.0026 |
|  | GO:0006303 | double-strand break repair via nonhomologous end joining | 1 | 0.0091 |
|  | GO:0072522 | purine-containing compound biosynthetic process | 2 | 0.0104 |
|  | GO:0006457 | protein folding | 2 | 0.0111 |
|  | GO:0055085 | transmembrane transport | 17 | 0.024 |
|  | GO:0022403 | cell cycle phase | 2 | 0.0281 |
|  | GO:0010388 | cullin deneddylation | 2 | 0.0281 |
|  | GO:0044848 | biological phase | 2 | 0.0281 |
|  | GO:0000085 | mitotic G2 phase | 2 | 0.0281 |
|  | GO:0051325 | interphase | 2 | 0.0281 |
|  | GO:0051329 | mitotic interphase | 2 | 0.0281 |
|  | GO:0051319 | G2 phase | 2 | 0.0281 |
|  | GO:0000338 | protein deneddylation | 2 | 0.0281 |
|  | GO:0042451 | purine nucleoside biosynthetic process | 2 | 0.0309 |
|  | GO:0046129 | purine ribonucleoside biosynthetic process | 2 | 0.0309 |
|  | GO:0008652 | cellular amino acid biosynthetic process | 6 | 0.0336 |
|  | GO:0042594 | response to starvation | 2 | 0.0377 |
|  | GO:0031667 | response to nutrient levels | 2 | 0.0377 |
|  | GO:0009267 | cellular response to starvation | 2 | 0.0558 |
|  | GO:0031669 | cellular response to nutrient levels | 2 | 0.0558 |
| 45 DPF | GO:0006817 | phosphate ion transport | 2 | 0.003 |
|  | GO:0010388 | cullin deneddylation | 3 | 0.0037 |
|  | GO:0000085 | mitotic G2 phase | 3 | 0.0037 |
|  | GO:1901566 | organonitrogen compound biosynthetic process | 11 | 0.006 |
|  | GO:0006457 | protein folding | 3 | 0.0103 |
|  | GO:0072522 | purine-containing compound biosynthetic process | 3 | 0.0162 |
|  | GO:0006914 | autophagy | 1 | 0.0164 |
|  | GO:0035335 | peptidyl-tyrosine dephosphorylation | 2 | 0.0191 |
|  | GO:0007166 | cell surface receptor signaling pathway | 2 | 0.0308 |
|  | GO:0055085 | transmembrane transport | 24 | 0.0317 |
|  | GO:0055114 | oxidation-reduction process | 43 | 0.0469 |
|  | GO:0042451 | purine nucleoside biosynthetic process | 2 | 0.0483 |
|  | GO:0046129 | purine ribonucleoside biosynthetic process | 2 | 0.0483 |
|  | GO:0010257 | NADH dehydrogenase complex assembly | 2 | 0.0496 |
|  | GO:0009812 | flavonoid metabolic process | 2 | 0.0498 |
| 60 DPF | GO:1901566 | organonitrogen compound biosynthetic process | 4 | 0.0036 |
|  | GO:0006457 | protein folding | 4 | 0.0046 |
|  | GO:0055085 | transmembrane transport | 11 | 0.0301 |
|  | GO:0008652 | cellular amino acid biosynthetic process | 2 | 0.0447 |
|  | GO:0055114 | oxidation-reduction process | 24 | 0.0535 |

^a^ The number of the genes enriched significantly in the corresponding GO term. ^b^ The *P* value <0.05 indicates the genes are significantly enriched in some GO term. 15, 30, 45 and 60 DPF indicate the seed samples at 15, 30, 45, and 60 days past flowering (DPF) from Line 8107, respectively
